# Systematic Analysis of the Binding Surfaces between tRNAs and Their Respective Aminoacyl tRNA Synthetase Based on Structural and Evolutionary Data

**DOI:** 10.3389/fgene.2017.00227

**Published:** 2018-01-08

**Authors:** Satoshi Tamaki, Masaru Tomita, Haruo Suzuki, Akio Kanai

**Affiliations:** ^1^Institute for Advanced Biosciences, Keio University, Tsuruoka, Japan; ^2^Systems Biology Program, Graduate School of Media and Governance, Keio University, Fujisawa, Japan; ^3^Faculty of Environment and Information Studies, Keio University, Fujisawa, Japan

**Keywords:** tRNA, Aminoacyl-tRNA synthetase, RNA–protein complex, interacting surface, molecular evolution

## Abstract

To determine the mechanism underlying the flow of genetic information, it is important to understand the relationship between a tRNA and its binding enzyme, a member of the aminoacyl-tRNA synthetase (aaRS) family. We have developed a novel method to project the interacting regions of tRNA–aaRS complexes, obtained from their three-dimensional structures, onto two-dimensional space. The interacting surface between each tRNA and its aaRS was successfully identified by determining these interactions with an atomic distance threshold of 3.3 Å. We analyzed their interactions, using 60 mainly bacterial and eukaryotic tRNA–aaRS complexes, and showed that the tRNA sequence regions that interacted most strongly with each aaRS are the anticodon loop and the CCA terminal region, followed by the D-stem. A sequence conservation analysis of the canonical tRNAs was conducted in 83 bacterial, 182 archaeal, and 150 eukaryotic species. Our results show that the three tRNA regions that interact with the aaRS and two additional loop regions (D-loop and TΨC-loop) known to be important for formation of the tRNA L-shaped structure are broadly conserved. We also found sequence conservations near the tRNA discriminator in the Bacteria and Archaea, and an enormous number of noncanonical tRNAs in the Eukaryotes. This is the first global view of tRNA evolution based on its structure and an unprecedented number of sequence data.

## Introduction

Transfer RNAs (tRNAs) are short noncoding RNAs, and typical cytoplasmic tRNAs are approximately 70–90 ribonucleotides long. The secondary structure of almost all tRNA molecules is a cloverleaf fold with four arms (the acceptor arm, D-arm, anticodon arm, and TΨ-arm) and is essentially conserved in the three domains of life, the Bacteria, Archaea, and Eukarya (Fujishima and Kanai, 2014). tRNAs adopt an L-shaped three-dimensional structure, with the anticodon region at one end and the CCA acceptor sequence at the other. They function as adaptor molecules, acting as the physical link between mRNAs and the amino acid sequences of proteins. Over the last decade, we have discovered novel disrupted tRNA genes encoding multiple-intron-containing tRNAs, split and tri-split tRNAs, and permuted tRNAs (Fujishima and Kanai, 2014). We have also investigated and discussed the evolution of the tRNA genes and their introns (Fujishima et al., 2010; Hamashima et al., 2016). Many noncoding RNAs act in concert with their binding proteins or specific RNA enzymes. For example, pre-tRNAs are processed by the endoribonuclease RNase P (Pannucci et al., 1999; Agrawal et al., 2014), an enzyme usually composed of an RNA–protein complex or a protein complex, such as that in the human mitochondrial system (Holzmann et al., 2008). The ribonucleotides in tRNAs are chemically modified by many modification enzymes (Björk and Hagervall, 2014; Hori, 2014) to generate mature, functional tRNAs. Therefore, the characterization of aminoacyl-tRNA synthetase (aaRS), one of the key tRNA enzymes directly involved in the translation step, is very important in understanding the evolution of the tRNA molecules and the genetic code system. aaRS is the enzyme that attaches a specific amino acid to the appropriate tRNA molecule. It is a multidomain protein that includes a catalytic domain and an anticodon-binding domain (Giegé and Springer, 2016). Some aaRSs also have additional domains such as an RNA-binding domain and an editing domain (Fukunaga and Yokoyama, 2005). The aaRSs are divided into six classes (classes Ia, Ib, Ic, IIa, IIb, and IIc) based on the structure of their catalytic sites (Giegé and Springer, 2016), although all the enzymes bind the highly conserved L-shaped structure of tRNAs. The tRNA molecule contains specific ribonucleotides, called “tRNA identity nucleotides” (Ibba et al., 1996; Giegé et al., 1998), that comprise the set of ribonucleotides responsible for the specific aminoacylation of tRNA with its cognate amino acid. Clarifying the structural biology of the tRNA–aaRS complexes is crucial for defining the similarities and/or differences among these complexes.

The structures of various RNA–protein complexes have already been determined with methods such as X-ray crystallography, nuclear magnetic resonance spectroscopy, and electron microscopy. As of May 16 2017, 2,207 RNA–protein complexes have been submitted to the Protein Data Bank (PDB) (Burley et al., 2017), and various bioinformatic algorithms and tools have been developed to utilize these structural data. For example, homology analyses at the structural level, based on root-mean-square deviations (Maiorov and Crippen, 1994), are used for the comparative analysis of molecular structures. Visualization and analytical methods based on contact map algorithms (Holm and Sander, 1996) are commonly used to determine the characteristics of protein–protein interactions, and these methods have also been applied to various RNA–protein complexes (Pietal et al., 2012). Machine learning techniques have been used to predict undetermined RNA–protein interactions (Fernandez et al., 2011; Muppirala et al., 2011; Akbaripour-Elahabad et al., 2016), where the interacting molecules in known structures are often used as the training and test data to achieve highly accurate predictions. Recent studies have also been successful in predicting interacting residues and ribonucleotides based on evolutionarily conserved sequence pairs (Li et al., 2014; Weinreb et al., 2016). Furthermore, the development of biological experimental protocols using high-throughput instruments, such as next-generation sequencing and mass spectrometry, has allowed the large-scale identification of interacting RNA and protein sequences (Scheibe et al., 2012). This knowledge has now been drawn together and published in several databases, including the Protein–RNA Interface Database (PRIDB) (Lewis et al., 2011), the Nucleic acid–Protein Interaction DataBase (NPIDB) (Kirsanov et al., 2013), and RBPmap (Paz et al., 2014). A comprehensive understanding of the characteristic RNA–protein interacting regions is especially important in the field of molecular biology, and a comparison of these interacting regions is essential for such an understanding.

Although various tRNA–aaRS structural data are available, in many cases, these data are only used for individual studies. However, it is necessary to analyze quantitatively and compare the overall interactions within these structures. Our purpose here was to determine the interaction characteristics of tRNA–aaRS complexes and the ribonucleotide conservation within each tRNA among the three domains of life. Therefore, in this study, we conducted a global analysis of almost all available tRNA–aaRS co-crystalized structures, and present a method to quantitatively compare the interaction information available for them. We detected common features and also heterogeneity among the tRNA–aaRS interactions across the three domains of life. Integrating these RNA–protein interaction data with a sequence conservation analysis, we demonstrate strikingly conserved regions in the tRNA–aaRS across all three domains of life, as well as domain of life-specific conserved tRNA positions. Together, these data provide a fundamental molecular resource for the fields of tRNA and aaRS research.

## Materials and methods

### Data sources

Three-dimensional structural data for 48 bacterial, two archaeal, and 10 eukaryotic tRNA–aaRS complexes were downloaded from the PDB (Burley et al., 2017) at http://www.rcsb.org/ on 5 May 2017. The amino acid sequences of the aaRSs, together with their annotations, were obtained from UniProtKB/Swiss-Prot (UniProt, 2015) using the ID cross-reference given in the PDB file. The original tRNA sequences of the species used in the PDB dataset were obtained from the Genomic tRNA Database (GtRNAdb) (Chan and Lowe, 2016). Mitochondrial tRNA–aaRS complexes were not considered in this analysis, because only one structure was available in the PDB. The information is summarized in Table 1 and Supplementary Table S1. In numbering the tRNA positions, the universal conventional tRNA position rule (Sprinzl et al., 1998) was used to specify the tRNA identity nucleotides in each tRNA and to compare the positions among the different sequences.

**Table 1:** Numbers of interacting ribonucleotides and amino acids in tRNA–aaRS complexes.

**aaRS class**	**tRNA type (anticodon)**	**Species (tRNA/aaRS)^§1^**	**PDB ID**	**No. of interacting ribonucleotides^§2^**	**No. of interacting amino acids^§3^**	**No. of interacting amino acids/No. of amino acids on surface**
**(A) BACTERIA**						
Ia	Cys (GCA)	*E. coli*	1U0B	16/74 (0.22)	25/461 (0.054)	25/249 (0.10)
Ia	Ile (GAT)	*S. aureus*	1FFY	27/75 (0.36)	39/917 (0.043)	39/446 (0.09)
Ia	Leu (TAA)	*E. coli*	4ARC	21/71 (0.30)	29/813 (0.036)	29/420 (0.07)
Ia	Leu (TAA)	*E. coli*	3ZJU	16/80 (0.20)	22/820 (0.027)	22/421 (0.05)
Ia	Leu (TAA)	*E. coli*	4ARI	18/80 (0.23)	22/821 (0.027)	22/413 (0.05)
Ia	Leu (TAA)	*E. coli*	4AQ7	25/79 (0.32)	33/860 (0.038)	33/435 (0.08)
Ia	Leu (TAA)	*E. coli*	3ZGZ	24/82 (0.29)	32/860 (0.037)	32/433 (0.07)
Ia	Leu (TAA)	*E. coli*	4AS1	16/83 (0.19)	22/812 (0.027)	22/416 (0.05)
Ia	Leu (TAA)	*E. coli*	3ZJT	20/83 (0.24)	23/820 (0.028)	23/420 (0.05)
Ia	Leu (TAA)	*E. coli*	3ZJV	15/85 (0.18)	20/813 (0.025)	20/416 (0.05)
Ia	Leu (n.d.)	*E. coli*	4CQN	26/82 (0.32)	43/860 (0.050)	43/418 (0.10)
Ia	Met (CAT)	*A. aeolicus*	2CT8	9/74 (0.12)	15/465 (0.032)	15/218 (0.07)
Ia	Met (CAT)	*A. aeolicus*	2CSX	9/75 (0.12)	13/464 (0.028)	13/219 (0.06)
Ia	Val (CAC)	*T. thermophilus*	1GAX	20/75 (0.27)	33/862 (0.038)	33/421 (0.08)
Ia	Val (CAC)	*T. thermophilus*	1IVS	19/75 (0.25)	30/862 (0.035)	30/422 (0.07)
Ib	Gln (CTG)	*E. coli*	4JXX	22/71 (0.31)	36/536 (0.067)	36/275 (0.13)
Ib	Gln (TTG)	*E. coli*	4JXZ	20/71 (0.28)	33/538 (0.061)	33/273 (0.12)
Ib	Gln (CTG)	*E. coli*	1EXD	24/73 (0.33)	43/529 (0.081)	43/264 (0.16)
Ib	Gln (CTG)	*E. coli*	1EUY	21/73 (0.29)	36/529 (0.068)	36/261 (0.14)
Ib	Gln (CTG)	*E. coli*	1GTR	24/74 (0.32)	34/529 (0.064)	34/266 (0.13)
Ib	Gln (CTG)	*E. coli*	1GTS	23/74 (0.31)	37/529 (0.070)	37/267 (0.14)
Ib	Gln (CTG)	*E. coli*	1QRS	27/74 (0.36)	43/529 (0.081)	43/265 (0.16)
Ib	Gln (CTG)	*E. coli*	1QRT	27/74 (0.36)	46/529 (0.087)	46/269 (0.17)
Ib	Gln (CTG)	*E. coli*	1QRU	27/74 (0.36)	51/529 (0.096)	51/271 (0.19)
Ib	Gln (CTG)	*E. coli*	1QTQ	22/74 (0.30)	30/529 (0.057)	30/268 (0.11)
Ib	Gln (CTG)	*E. coli*	2RD2	19/74 (0.26)	34/529 (0.064)	34/265 (0.13)
Ib	Gln (CTG)	*E. coli*	2RE8	20/74 (0.27)	32/529 (0.060)	32/261 (0.12)
Ib	Gln (CTG)	*E. coli*	1ZJW	21/74 (0.28)	36/529 (0.068)	36/265 (0.14)
Ib	Gln (CTG)	*E. coli*	1O0B	23/74 (0.31)	38/529 (0.072)	38/264 (0.14)
Ib	Gln (CTG)	*E. coli*	1O0C	21/74 (0.28)	37/529 (0.070)	37/270 (0.14)
Ib	Glu (CTG)	*T. martima*	3AKZ	23/74 (0.31)	35/463 (0.076)	35/237 (0.15)
Ib	Glu (CTC)	*T. thermophilus*	1N77	16/75 (0.21)	32/468 (0.068)	32/253 (0.13)
Ib	Glu (CTC)	*T. thermophilus*	1N78	19/75 (0.25)	35/468 (0.075)	35/251 (0.14)
Ib	Glu (CTC)	*T. thermophilus*	2CV1	15/75 (0.20)	32/468 (0.068)	32/253 (0.13)
Ib	Glu (CTC)	*T. thermophilus*	2CV2	17/75 (0.23)	32/468 (0.068)	32/246 (0.13)
Ib	Glu (CTC)	*T. thermophilus*	2DXI	18/75 (0.24)	32/468 (0.068)	32/251 (0.13)
Ib	Glu (CTC)	*T. thermophilus*	2CV0	19/75 (0.25)	34/468 (0.073)	34/252 (0.13)
Ib	Glu (CTC)	*T. thermophilus*	1G59	20/75 (0.27)	34/468 (0.073)	34/247 (0.14)
Ic	Tyr (GTA)	*T. thermophilus*	1H3E	19/84 (0.23)	22/854 (0.026)(dimer)	22/429 (0.05)(dimer)
IIa	His (GTG)	*T. thermophilus*	4RDX	14/77 (0.18)	22/409 (0.054)	22/237 (0.09)
IIa	Pro (CGG)	*T. thermophilus*	1H4Q	11/67 (0.16)	21/465 (0.045)	21/420 (0.05)
IIa	Ser (GGA)	*T. thermophilus*	1SER	13/65 (0.20)	12/372 (0.032)	12/375 (0.03)
IIa	Thr (CGT)	*E. coli*	1QF6	18/76 (0.24)	29/641 (0.045)	29/331 (0.09)
IIb	Asp (GTC)	*T. thermophilus*	1EFW	11/73 (0.15)	13/580 (0.022)	13/324 (0.04)
IIb	Asp (GTC)	*S. cerevisiae/E. coli*	1IL2	22/75 (0.29)	35/585 (0.060)	35/323 (0.11)
IIb	Asp (GTC)	*E. coli*	1C0A	21/77 (0.27)	33/585 (0.056)	33/319 (0.10)
IIc	Phe (GAA)	*T. thermophilus*	2IY5	24/76 (0.32)	17/672 (0.025) (alpha × 2) 15/1,562 (0.010) (beta × 2)	32/1,027 (0.03) (dimer × 2)
IIc	Phe (GAA)	*T. thermophilus*	1EIY	16/76 (0.21)	11/690 (0.016) (alpha × 2) 10/1,570 (0.006) (beta × 2)	21/1,048 (0.02) (dimer × 2)
**aaRS class**	**tRNA type (anticodon)**	**Species**	**PDB ID**	**No. of interacting ribonucleotides^§2^**	**No. of interacting amino acids^§3^**	**No. of interacting amino acids/No. of amino acids on surface**
**(B) ARCHAEA**						
Ia	Leu (CAA)	*P. horikoshii*	1WZ2	16/88 (0.18)	27/948 (0.028)	27/463 (0.06)
Ic	Tyr (GTA)	*M. jannaschii*	1J1U	10/75 (0.13)	18/598 (0.030)(dimer)	18/282 (0.03)(dimer)
**aaRS class**	**tRNA type(anticodon)**	**Species (tRNA/aaRS)^§1^**	**PDB ID**	**No. of interacting ribonucleotides^§2^**	**No. of interacting amino acids^§3^**	**No. of interacting amino acids/No. of amino acids on surface**
**(C) EUKARYA**						
Ia	Arg (ICG)	*S. cerevisiae*	1F7U	21/75 (0.28)	32/606 (0.053)	32/297 (0.10)
Ia	Arg (ICG)	*S. cerevisiae*	1F7V	15/72 (0.21)	23/606 (0.038)	23/301 (0.08)
IIc	Trp (CCA)	*B. taurus/H. sapiens*	2AKE	8/72 (0.11)	16/746 (0.021)(dimer)	16/346 (0.05)(dimer)
Ic	Trp (CCA)	*B. taurus/H. sapiens*	2DR2	13/75 (0.17)	22/746 (0.029)(dimer)	22/348 (0.06)(dimer)
IIa	Gly (CCC)	*H. sapiens*	5E6M	22/74 (0.30)	31/1,038 (0.029)(dimer)	31/497 (0.06)(dimer)
IIa	Gly (CCC)	*H. sapiens*	4QEI	19/69 (0.28)	30/1,124 (0.027)(dimer)	30/591 (0.05)(dimer)
IIa	Gly (CCC)	*H. sapiens*	4KR2	17/69 (0.25)	23/916 (0.025)(dimer)	23/430 (0.05)(dimer)
IIa	Gly (CCC)	*H. sapiens*	4KR3	20/70 (0.29)	30/932 (0.032)(dimer)	30/436 (0.07)(dimer)
IIb	Asp (GTC)	*S. cerevisiae*	1ASY	14/75 (0.19)	32/490 (0.065)	32/426 (0.08)
IIb	Asp (GTC)	*S. cerevisiae*	1ASZ	15/75 (0.20)	31/490 (0.063)	31/423 (0.07)

§1 *If two species names occur in one column, the first represents the organism from which the tRNA is derived and the second the organism from which the aaRS is derived*.

§2 *Ratio of the number of interacting ribonucleotides to the total number of ribonucleotides used in the structural analysis (ratio)*.

§3 *Ratio of the number of interacting amino acids to the total number of amino acids used in the structural analysis (ratio)*.

*n.d.: not determined*.

For the evolutionary conservation analysis, 83 bacterial, 182 archaeal, and 150 eukaryotic tRNA sequences, together with their secondary structures, were obtained from GtRNAdb (Chan and Lowe, 2016) on 15 April 2017. If more than two identical species were found in GtRNAdb, the tRNA data for the most recently registered genome were used for the analysis. The National Center for Biotechnology Information (NCBI) (O'Leary et al., 2016) reference genome list (downloaded on 26 July 2016 from ftp://ftp.ncbi.nlm.nih.gov/genomes/GENOME_REPORTS/prok_reference_genomes.txt) was used to select the representative bacterial genomes. For detailed information, see Supplementary Table S2.

### Routine software used in this study

The Biopython package version 1.6 (Cock et al., 2009) was used for preprocessing and analysis of the structural data. Because some of the tRNA sequences in the PDB files differ from the original sequences, in terms of either deleted or substituted ribonucleotides, a BLAST (Camacho et al., 2009) search of GtRNAdb was performed using an E-value cut-off of 1e−25, to determine the original tRNA sequences in each dataset. ClustalW (Larkin et al., 2007) was used to align the sequence of the crystallized molecule with the original tRNA sequence to allow insertion of gaps. tRNAscan-SE version 1.3.1 was used to predict the secondary structures to determine the tRNA anticodons. We defined the amino acid residues present on the aaRS surface as those meeting the following criterion:

relative accessible surface area (RSA) < 20%

The RSA was calculated by DSSP (Kabsch and Sander, 1983; Touw et al., 2015) using the accessible surface area prediction method of Rost and Sander (Rost and Sander, 1994).

### Distance calculations within tRNA–aaRS complexes

Before any distances were calculated, the atomic coordinates of the tRNA–aaRS complexes were obtained from the corresponding PDB files. In this study, atom pairs of tRNA and aaRS within a distance of 3.3 Å were defined as interacting. The distance between a tRNA ribonucleotide and an aaRS amino acid was calculated with the following equation:

distance (X,Y) = inf{d(x,y) | x ∈ X, y ∈ Y}

where X represents all the atoms included in an amino acid of aaRS, Y represents all the atoms included in a ribonucleotide of tRNA, and *d* is the Euclidean distance. The interacting atoms and amino acid residues calculated in this study are summarized in Supplementary Table S3.

### Determination and visualization of the three-dimensional interacting structures of the tRNA–aaRS complexes

The interacting regions of the tRNA–aaRS complexes were determined with a Python script using Biopython (Cock et al., 2009). The three-dimensional structural data for each tRNA and tRNA–aaRS complex were visualized with the Python interface of Chimera (Pettersen et al., 2004). The interacting region of the tRNA–aaRS complex was determined based on the calculation of the Euclidean distances, as described above. Using consecutive visualizations based on multiple distance cut-off points, we determined the distance threshold that met most of the following criteria: (a) minimized the distances between the components of the tRNA–aaRS complex; (b) maximized the numbers of tRNA positions known as tRNA identity nucleotides within the region; and (c) included only one strand of tRNA regions forming a stem. In some cases, biological assemblies had been created for the aaRS complexes, which act as multimeric proteins, based on the BIOMT records in the PDB files. When more than two biological assemblies were registered in the structure file, the first biological assembly declared in the BIOMT annotation of the PDB file was used for the analysis. If more than two tRNAs were included in the assembly, one of the tRNA molecules in the assembly was selected and used for the analysis, because the interaction patterns between the two tRNA were almost the same.

### Systematic analysis of the interacting region of each tRNA–aaRS complex

Based on the output of the tRNAscan-SE secondary structure prediction file, the tRNAs were divided broadly into 15 sequence regions (S1–S15) based on their cloverleaf structure. For each tRNA sequence region, we calculated the normalized number of interacting amino acids, as follows. First, the sum of the number of interacting amino acids per ribonucleotide within each sequence region was calculated. The numbers were then normalized to the number of ribonucleotides in the sequence region. tRNA positions that were undetermined in the PDB file were excluded from the calculation. When a tRNA sequence region included 25% or more ribonucleotides that were structurally undetermined in the PDB file, the sequence region was regarded as undetermined. A representative tRNA dataset with a minimum number of undetermined positions (up to three, corresponding to each amino acid) was selected for further analysis. To handle the datasets with undetermined tRNA sequence regions, the R (version 3.4.0) function cor() with option “pairwise.complete.obs” in R was used to calculate the correlation distance (one minus the Pearson product-moment correlation coefficient) between the data. The interacting features were hierarchically clustered using the furthest neighbor method.

### Analysis of evolutionarily conserved regions in the tRNA sequences

For each species, a unique tRNA sequence was selected and used for the analysis, regardless of its tRNA copy number in the genome. Using tRNA information from GtRNAdb, the tRNA that fulfilled the following criteria was extracted: (a) was not a pseudogene; (b) had a structure that followed the universal tRNA positioning rules (Sprinzl et al., 1998); and (c) did not contain characters other than “A, U, G, and C,” because letters “R” and “N” occur in some tRNA sequences. The tRNA sequences corresponding to the same amino acid were aligned according to their secondary structures. The aligned tRNA sequences used for this analysis are summarized in Table 2. From the aligned sequences, a conservation score for each tRNA position was calculated using Claude Shannon's entropy:

$$H\;\left(l\right)\;=\;-\Sigma f\left(b,l\right)\log_{2}f\left(b,l\right),$$

where H(*l*) is the entropy at tRNA position *l, b* represents each ribonucleotide (A, U, G, and C), and *f* (*b,l*) is the frequency of base *b* observed at position *l*. Gaps in the alignment were skipped in calculating the entropy for each position. The entropy value was divided into nine ranks to measure the degree of conservations. tRNA positions with the prefix “e” (part of the V-arm) and the CCA terminal sequence region were excluded from the analysis. The calculated conservation scores were mapped to the tRNA positions based on the universal tRNA positioning rules. The arrangement and color scheme used to map the conservation scores were according to ConSurf (Ashkenazy et al., 2016).

**Table 2:** Number of canonical and noncanonical tRNAs used in this study.

**tRNA type**	**Bacteria (*****n*** = **83)**		**Archaea (*****n*** = **182)**		**Eukaryotes (*****n*** = **150)**	
	**No. of canonical tRNA**	**No. of non-canonical tRNA**	**No. of canonical tRNA**	**No. of non-canonical tRNA**	**No. of canonical tRNA**	**No. of non-canonical tRNA**
Ala	325	2	583	9	4,139	11,391
Arg	417	17	832	21	3,328	4,168
Asn	189	1	208	3	2,292	1,125
Asp	188	2	208	7	2,188	663
Cys	91	1	249	48	1,799	1,135
Gln	185	5	356	1	2,125	1,858
Glu	232	1	379	7	4,150	6,589
Gly	365	5	569	12	3,629	12,297
His	97	1	179	4	1,374	534
Ile	194	1	206	5	1,870	1,414
Leu	477	3	691	215	1,911	3,875
Lys	251	1	355	4	3,881	4,712
Met	389	12	581	2	1,903	1,847
Phe	131	1	207	5	1,234	710
Pro	224	3	490	11	1,423	2,567
Ser	337	23	539	165	2,013	3,540
Thr	295	2	520	29	2,840	1,786
Trp	93	0	181	1	952	1,007
Tyr	130	3	181	6	1,079	1,181
Val	307	2	537	25	1,763	4,570

*See Figure 3 and Supplementary Table S2 for information on canonical tRNAs*.

## Results and discussion

### Determination of tRNA–aaRS interacting regions

In this study, we defined the interacting regions of the tRNA–aaRS complexes as follows. A tRNA and amino acid were considered to interact when the atoms comprising the ribonucleotides and amino acids occurred within a specific distance (the Euclidean distance between the atoms). We established the appropriate distance threshold by taking the bacterial tRNA^Gln^–GlnRS complex as an example. The tRNA^Gln^–GlnRS interacting surface based on various distance thresholds is shown in Supplementary Figure S1 (for tRNA ribonucleotides) and Supplementary Figure S2 (for aaRS amino acids). The Euclidean distance thresholds tested ranged from 2.0 to 5.0 Å in 0.1 Å increments and the atoms involved in each interacting region were analyzed. As a result, 3.3 Å was selected as the threshold interaction distance. This threshold was also suitable for specifying the interacting surfaces of other tRNA–aaRS complexes (Supplementary Figure S3) from both sides: from the ribonucleotides of the tRNAs and from the amino acids of the aaRSs. When distances smaller than 3.3 Å were used as the threshold, the distance threshold did not meet the necessary criteria for neighboring molecules (see the section “Determination and visualization of the three-dimensional interacting structures of the tRNA–aaRS complexes” in the Materials and Methods). When distance thresholds greater than 3.3 Å were used, molecules other than those involved in the interacting surface were included.

**Figure 1:**
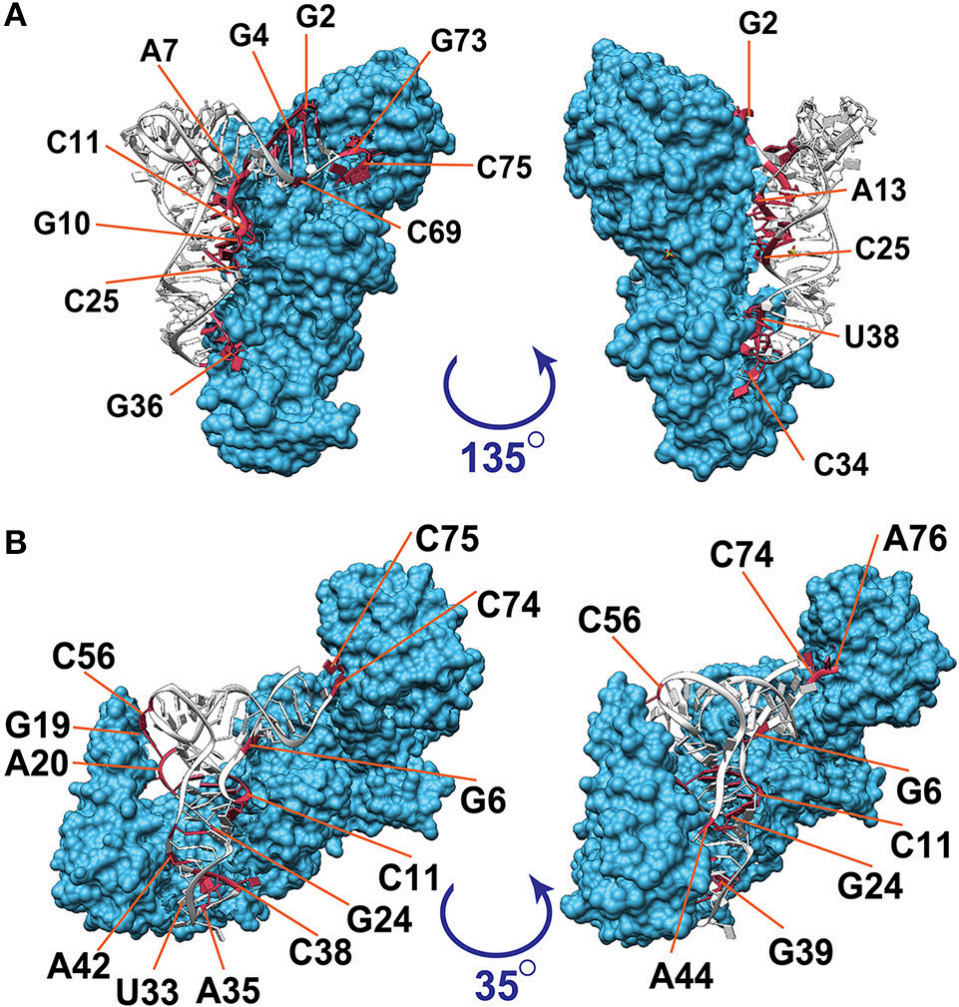
Visualization of the interacting ribonucleotides in tRNA–aaRS complexes. Three-dimensional illustrations of **(A)** the tRNA^Gln^–GlnRS complex (PDB ID: 1exd) and **(B)** the tRNA^Val^–ValRS complex (PDB ID: 1gax) are shown. Amino acid residues in aaRS are colored blue; nucleotides in tRNA that are within 3.3 Å of aaRS are colored red; and other nucleotides in tRNA are colored white. Numbers following the base symbols (A, U, G, and C) indicate the nucleotide positions in tRNAs. Nucleotide numbers in this figure are based on the universal conventional numbering system for tRNA positions (Sprinzl et al., 1998).

**Figure 2:**
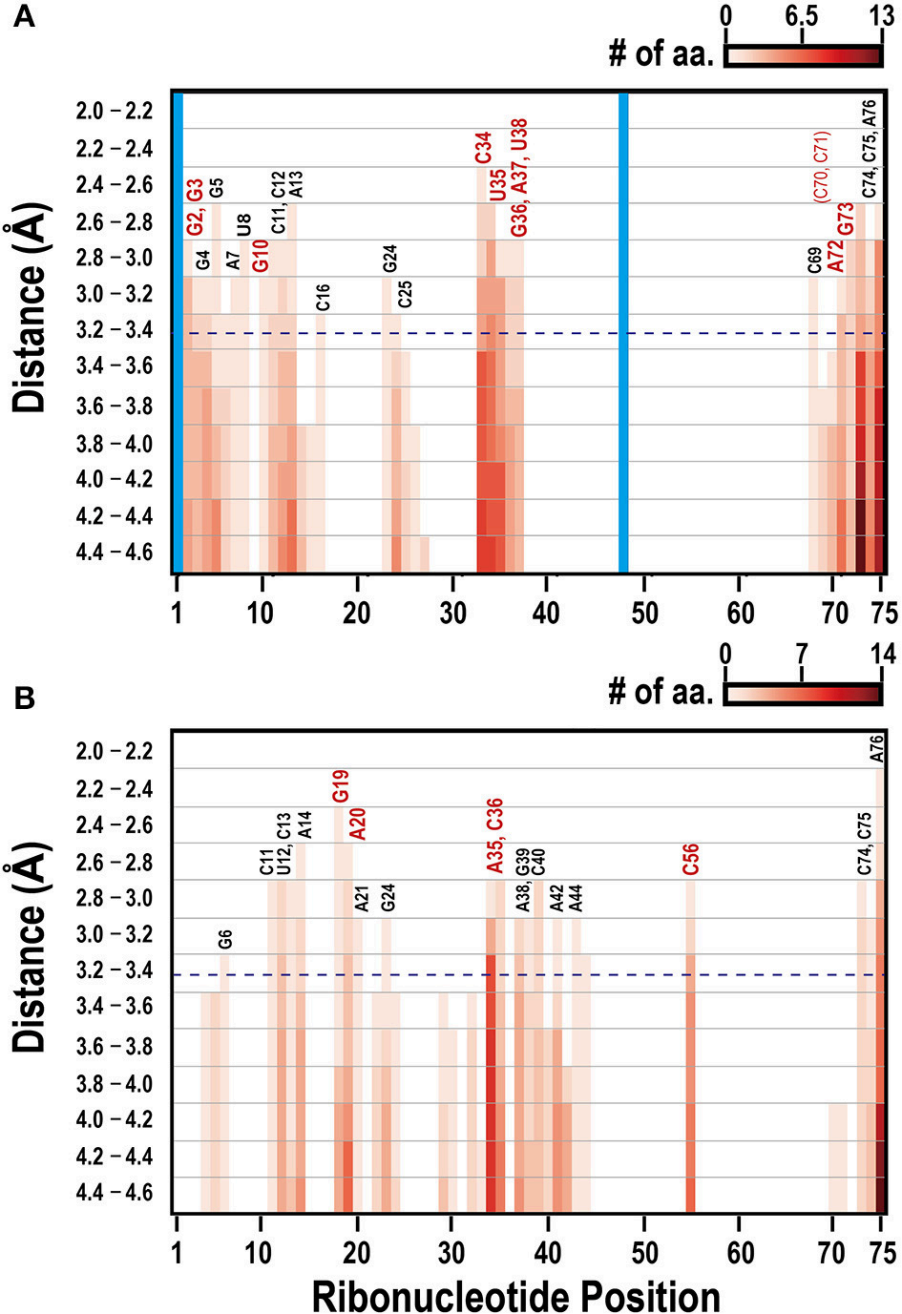
Mapping the interacting ribonucleotides in tRNA–aaRS complexes in bacterial tRNA–aaRS complexes. Two-dimensional maps of the interacting ribonucleotides in the *E. coli* tRNA^Gln^–GlnRS complex **(A)** and the *T. thermophilus* tRNA^Val^–ValRS complex **(B)**. The *x*-axis indicates the ribonucleotide positions in tRNAs from the 5′ end, and the *y*-axis indicates the Euclidean distance between the nucleotide and an amino acid. Blue dashed horizontal line shows the position of the threshold distance of 3.3 Å. Colors (white to dark red) of the bar correspond to the cumulative numbers of amino acid residues involved in the interaction with tRNA. Structurally undetermined ribonucleotides and gapped regions, determined from an alignment with the original tRNA sequence, are shown in the vertical blue bar. Numbers above the bars show the universal conventional tRNA positions, with conventional base symbols. Important ribonucleotides required for tRNA functions, such as the tRNA identity nucleotides and those affecting aminoacylation efficiency, are shown in red (see text for details).

Figure 1 illustrates the interacting tRNA molecules in the tRNA^Gln^–GlnRS and tRNA^Val^–ValRS complexes based on a threshold of 3.3 Å. The interacting surfaces of the tRNAs and aaRSs were identified in these tertiary structures. We then analyzed these interacting regions together with the tRNA positions (Figure 2) to generate a two-dimensional map of the interacting regions, which is also illustrated in Figure 1. In Figure 2A, the anticodon loop region (C34–G36 in Figure 1A) and the CCA region (G73–A76 in Figure 1A) of *Escherichia coli* tRNA^Gln^ interact with GlnRS at a distance of ~2.7 Å. When 3.3 Å was selected as the threshold distance, ribonucleotides including the tRNA identity nucleotides G2, G3, G10, C34, U35, G36, A37, U38, A72, and G73 (Figure 2A, red-colored ribonucleotides) were included in the interacting region. Ribonucleotides G2 and G3, which occur in the two ribonucleotide pairs G2:C70 and G3:C71 and are reported to correspond to tRNA^Gln^ identity nucleotides, were included in the interacting region. Although it has been reported that these pairs are tRNA identity nucleotides, this result suggests that ribonucleotides G2 and G3 play an even more important role in the interaction of the complex components. Similarly, when 3.3 Å was selected as the threshold for the tRNA^Val^–ValRS complex, as shown in Figure 2B, positions G19, A20, and C56 (also see Figure 1B), which are known to strongly influence the *k*_cat_ value of tRNA aminoacylation, were included in the interacting region. The reported tRNA identity nucleotides of *Thermus thermophilus* tRNA^Val^ include the anticodon ribonucleotides A35 and C36 (Fukai et al., 2003), and these two ribonucleotides are also included in the interacting region. Ribonucleotide C34 is located further from aaRS than the other two ribonucleotides, although the difference was only 1 Å. It has been reported that C34 is not recognized by ValRS (Fukai et al., 2003), supporting the proposition that the distance threshold should be less than 4.2 Å (Figure 2B). Because C34 corresponds to the third position of the codon, and because the third position of Val includes all four ribonucleotides (A, U, G, and C) in the standard bacterial codon table, the observation that ValRS recognizes the two non-variable ribonucleotides in the anticodon region is convincing. Most importantly, these results suggest that the interacting region is involved in the function of the tRNA^Val^–ValRS complex. Therefore, the interacting region identified with the 3.3 Å threshold is suitable for drawing conclusions about the tRNA–aaRS complex.

An increase in the interacting distance threshold from 3.3 Å had a relatively small effect on the number of ribonucleotides found to be included in the interacting region (Figure 2); thus, we assume that the biological conclusions drawn using the 3.3 Å threshold are robust to some extent. Furthermore, the average distance between the alpha carbons of the amino acids is approximately 3.8 Å (Podtelezhnikov and Wild, 2008), and increasing the threshold distance beyond this value may increase the risk of including amino acids outside of the interacting region. The distance thresholds used in recent studies to determine residue–ribonucleotide interactions were 2.7–3.9 Å (Jones et al., 2001), 3.5 Å (Li et al., 2014), and 5 Å (Ren and Shen, 2015), and our distance threshold of 3.3 Å is similar to those values.

### Systematic analysis of the interacting regions of tRNA–aaRS complexes

**Figure 3:**
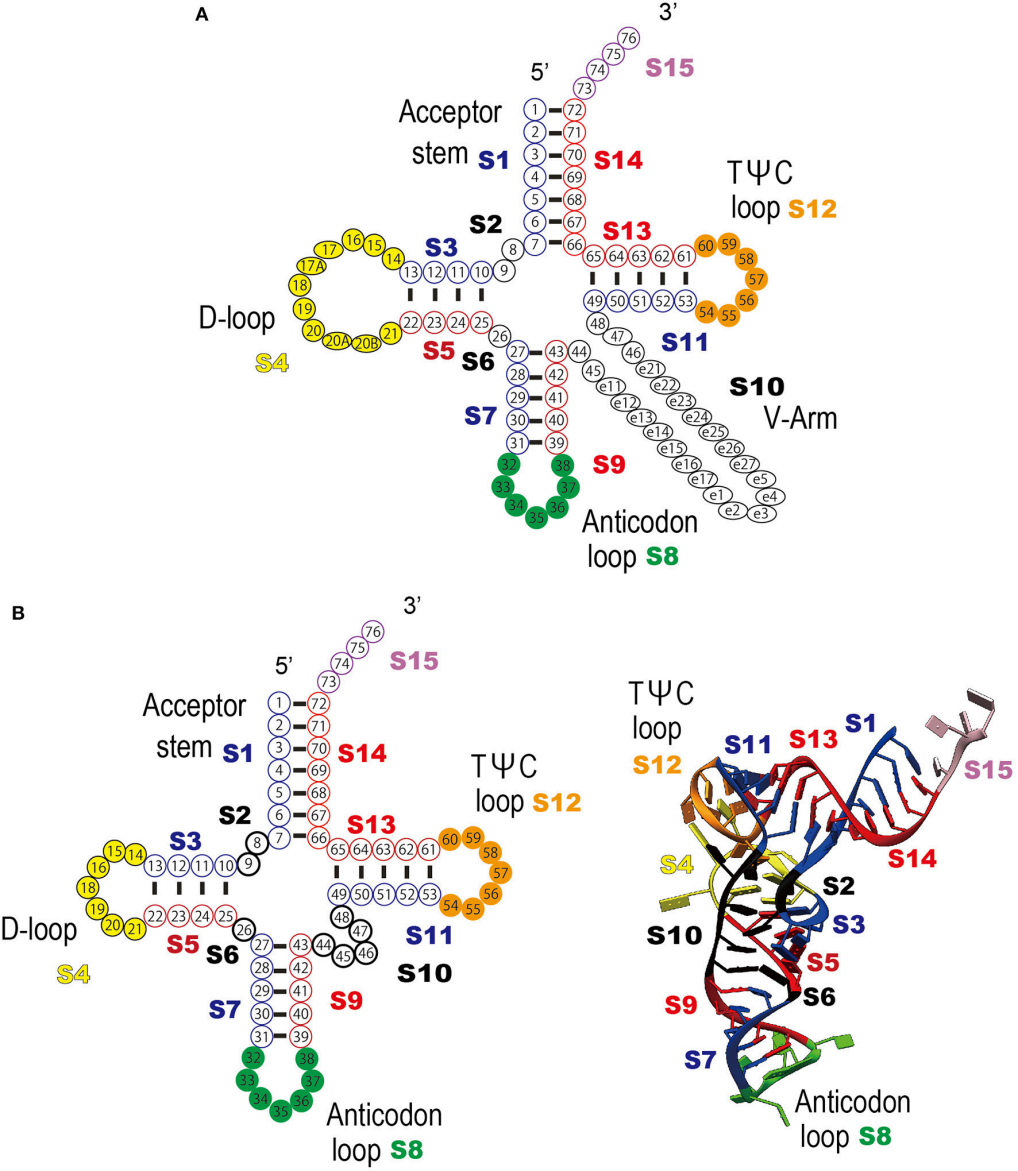
Definition of the tRNA numbering rule and the sequence regions used in the clustering analysis. **(A)** Schematic representation of the tRNA secondary structure and each tRNA sequence region (S1–S15) are shown; acceptor stem (S1 and S14), D-stem (S3 and S5), D-loop (S4), anticodon stem (S7 and S9), anticodon loop (S8), TΨC-stem (S11 and S13), variable region (S10), TΨC-loop (S12), 3′-terminal CCA region (S15), and the residues between the stems (S2 and S6). Each sequence region is shown in a different color. Ribonucleotide positions are defined as follows; S1 (positions 1–7), S2 (positions 8–9), S3 (positions 10–13), S4 (positions 14–21), S5 (positions 22–25), S6 (position 26), S7 (positions 27–31), S8 (positions 32–38), S9 (positions 39–43), S10 (positions 44–48), S11 (positions 49–53), S12 (positions 54–60), S13 (positions 61–65), S14 (positions 66–72), and S15 (positions 73–76). The figure was adapted from Sprinzl et al. (1998), with some modifications **(B)** Example of the universal tRNA numbering rule applied to tRNA^Val^ (PBD ID: 1gax) (see also Figure 1B). Left column: schematic representation of the tRNA clover leaf structure and bipartite tRNA sequence regions. Right column: schematic representation of the tRNA tertiary structure and the bipartite tRNA sequence regions.

**Figure 4:**
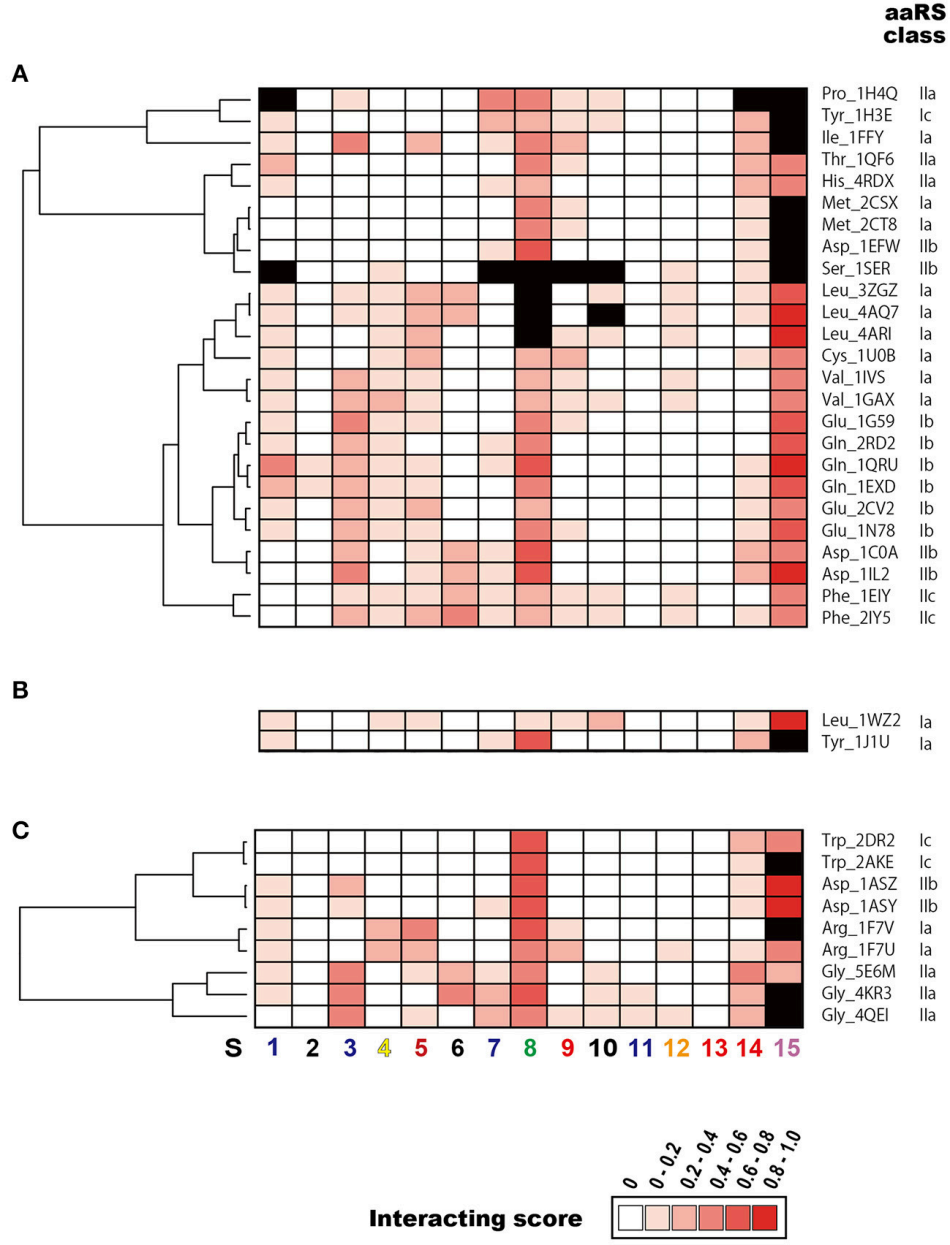
Clustering analysis of tRNA sequence regions that interact with aaRS. Heatmap of the interacting scores (normalized number of amino acids that interact with each tRNA sequence region) in **(A)** Bacteria, **(B)** Archaea, and **(C)** Eukarya are shown. Red colors in each cell indicate the rank of the interacting score. Note that the maximum interacting amino acid number (i.e., interacting score = 1.0) differs among the domains: 4.25 for Bacteria, 3.25 for Archaea, and 4.0 for Eukarya. Structurally undetermined tRNA sequence regions (see Materials and Methods) are shown in black. Dendrogram on the left side of each heatmap indicates similarities among tRNAs. The numbers shown at the bottom of the figure represent the sequence regions described in Figure 3.

To determine the common features and the heterogeneity of the molecular interactions in the tRNA–aaRS complexes, the tRNAs were divided broadly into 15 sequence regions based on their cloverleaf structure (Figure 3). Each ribonucleotide was determined according to the universal tRNA positioning rules (Sprinzl et al., 1998) (Figure 3A). An example of the sequence regions of the tRNA^Val^ cloverleaf structure and its L-shaped structure are shown (Figure 3B). The interacting score for each sequence region was calculated for 14 kinds of the 48 representative bacterial tRNA–aaRS complexes (Figure 4A), two kinds of the two archaeal tRNA–aaRS complexes (Figure 4B), and four kinds of the 10 eukaryotic tRNA–aaRS complexes (Figure 4C). A clustering analysis of the interacting scores revealed features of the tRNA stems and loop regions at a glance, so that the data could be readily compared between different complexes.

The interacting ribonucleotides of the tRNAs were similar among Bacteria, Archaea, and Eukarya (see also Supplementary Figure S4). The most-interactive regions in the tRNA molecules were the anticodon loop (S8) and the CCA region (S15). The interaction involved the anticodon region in all complexes, except in two cases: in the first, no interaction was observed in one (4cqn) of the bacterial Leu complexes, and in the other case, the anticodon region was not determined in the crystal structures of the bacterial Leu (4arc, 3zjt, 4as1, 3zjv, 4ari, 3zju, 3zgz, and 4aq7) and Ser (1ser) complexes. It has been reported that bacterial tRNA^Leu^, tRNA^Ser^, and tRNA^Ala^ do not contain tRNA identity nucleotides in the anticodon loop region (Giegé et al., 1998). The interaction was observed in all the complexes with structurally determined tRNA CCA regions. Because the structural data for the CCA region was largely or completely missing in the datasets for the bacterial Asp (1efw), Ile (1ffy), Met (2ct8 and 2csx), Pro (1h4q), and Ser (1ser) samples and the eukaryotic Arg (1f7v), Gly (4kr2, 4kr3, and 4qei), and Trp (2ake) samples, the interactions of the CCA regions in these complexes could not be determined. Followed by the CCA region and anticodon loop region, one side of the D-stem (S3) region and one side of the acceptor stem region (S14) of many tRNAs interact with aaRSs. These characteristic interactions are basically conserved among all the domains of life, but it is difficult to discuss the generality of the interaction patterns in the Archaea because only two tRNA–aaRS complexes are available. The variable region of the V-arm (positions indicated with an “e” in Figure 3A) was not evaluated in the current study, because comprehensive and comparative analyses of this region are difficult. Of the individual long-V-arm-containing tRNAs (i.e., bacterial tRNA^Tyr^, tRNA^Ser^, and tRNA^Leu^), tRNA^Tyr^ (PDB ID; 1h3e) possesses a V-arm containing 14 ribonucleotides, 4 (28.6%) of which interact with the TyrRS. In the case of tRNA^Ser^ (PDB ID; 1ser), although the crystal structures of 8 ribonucleotides in its V-arm (containing 22 ribonucleotides) have not been determined, 6 of the 14 ribonucleotides (42.9%) that were structurally determined interact with SerRS. Finally, tRNA^Leu^ (PDB ID; 4arc) possesses a V-arm containing 14 ribonucleotides, 2 (14.3%) of which interact with LeuRS. These results showed that the long-V-arm-containing tRNAs interact with each cognate aaRS via the long V-arm region, and this characteristic is unique to these tRNAs.

The 5′ half of each tRNA molecule contains more interacting ribonucleotides than the 3′ half of the molecule. When the interactions within the tRNA–aaRS complexes were considered, sequence regions S2, S3, S5, and S6 projected onto the interacting surface (Figure 3, see also Figure 1). One strand (S3) of the D-stem interacts with a number of amino acids (in 15 of the 23 bacterial tRNAs and six of the 10 eukaryote tRNAs). As mentioned above, because tRNAs form three-dimensional L-shaped structures to interact with aaRSs, this stem region is expected to project into the interacting surface when the anticodon and CCA regions interact with aaRS. Some tRNA–aaRS complexes, such as the bacterial Leu, Tyr, Val, Phe, and Ser complexes and the eukaryotic Gly complex, had relatively high scores for the interacting ribonucleotides in the 3′ half of the tRNA molecule. For example, the interacting region in the TΨC-arm (S12) of bacterial tRNA^Val^ interacts with the tRNA-binding arm (coil-to-coil domain) in ValRS (Supplementary Figure S5).

aaRSs can be categorized into six classes (Ia, Ib, Ic, IIa, IIb, and IIc) by the sequence homology of their catalytic domains. The dendrogram in the Figure 4 shows the aaRS classes classified according to the similarities in their patterns of interaction with their tRNAs. In Bacteria, these six classes of aaRSs occur in a mosaic pattern, in which the tRNA–aaRS complexes corresponding to each class are intermingled (Figure 4A). This result suggests that the variations in the interaction features of tRNA–aaRS do not always depend on the aaRS class in Bacteria. The numbers of interacting tRNA ribonucleotides and aaRS amino acids are summarized in Table 1. In some cases, the numbers of interacting molecules (ribonucleotides or amino acid residues) in a distinct structural experiment differed, even for the same tRNA–aaRS complex within the same species. This could be because of sequence variations in the aaRS and/or tRNA, different sets of small ligands present during crystallization, or additional differences in crystallization or other conditions that lead to different conformations of the complex in the crystalline lattice that are likely of functional relevance.

Therefore, we calculated the average numbers of interacting molecules in all the available tRNA–aaRS complexes (bacteria, 48; archaea, two; and eukaryotes, 10). The average number of interacting ribonucleotides and amino acids were: 19.5 ± 4.6 ribonucleotides and 30.7 ± 8.6 amino acids in Bacteria, 13.0 ± 3.0 ribonucleotides and 22.5 ± 4.5 amino acids in Archaea, and 16.4 ± 4.0 ribonucleotides and 27.0 ± 5.3 amino acids in Eukarya. These numbers correspond to approximately 25.0% of the tRNA molecule sequence and 4.9% of the aaRS molecule sequence. When we compared the ratio of the interacting amino acid residues to the amino acid residues present on the aaRS surface, 9.3% of residues were involved in the interaction. Although the aaRS molecule is much larger than the tRNA molecule, the proportion of interacting ribonucleotides in the tRNA is much greater than the proportion of interacting amino acids in the protein. Precise information on each interacting ribonucleotide and amino acid residue is available in Supplementary Table S3.

### Comparative analysis of the interacting regions and evolutionarily conserved regions in tRNA–aaRS complexes

**Figure 5:**
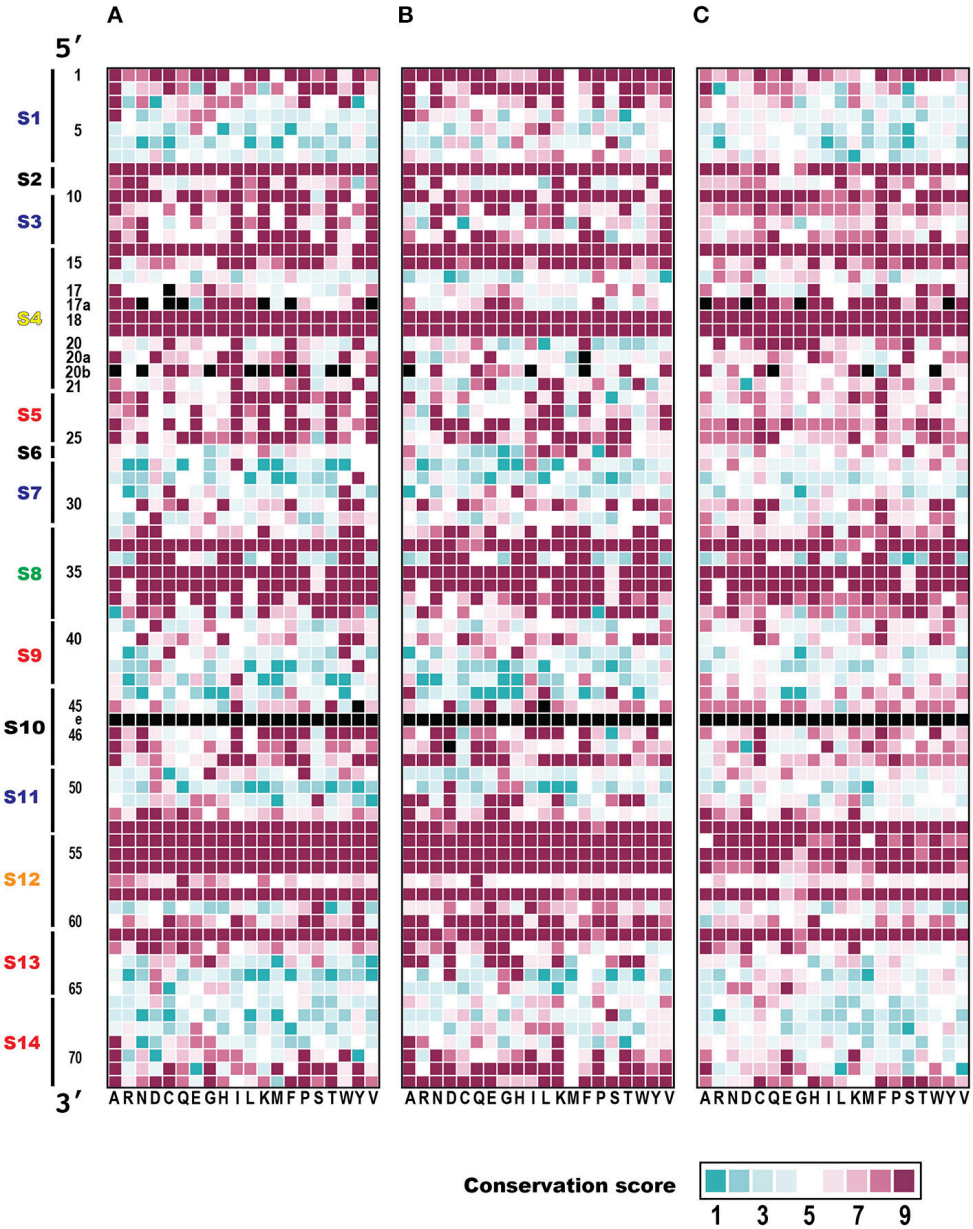
Evolutionary conservation analysis of ribonucleotide positions of tRNAs. For each tRNA position, the conservation score based on Claude Shannon's entropy in **(A)** Bacteria (83 species), **(B)** Archaea (182 species), and **(C)** Eukarya (150 species) are shown as nine ranks, ranging from variable (rank 1: cyan) to conserved (rank 9: magenta). The X row lists the 20 families of isoaccepting tRNAs (with their corresponding amino acids displayed in the single-letter code). The Y column shows the tRNA ribonucleotide positions based on the universal conventional tRNA positions (Sprinzl et al., 1998), and the tRNA sequence regions described in Figure 3. Missing ribonucleotides in the tRNA positions (at positions 17, 17a, 20a, 20b, 45, 46, and 47) and tRNA positions with prefix ‘e’ (Sprinzl et al., 1998) (part of V-arm) were excluded from the analysis and the cells are colored black. Note that the conservation score was calculated for each position of all tRNAs according to their cognate amino acids from a number of each species, but not between tRNA with different cognate amino acids.

To determine how the interacting surfaces of tRNAs have been conserved during evolution, we performed an exhaustive sequence conservation analysis of the bacterial, archaeal, and eukaryotic tRNAs (Figure 5). The CCA terminal sequence region was not used in this analysis because it is not always encoded in the tRNA gene. Figure 5 shows the overall characteristics of the tRNA interactions, in which certain areas of the tRNA molecule are broadly conserved among the 20 types of tRNAs: all three loop regions (D-loop, S4; anticodon loop, S8; and TΨC-loop, S12) and two of the eight stem regions (D-stem, S3 and S5) are highly conserved (also see Supplementary Figure S6).

Taking the bacteria as an example, all the regions in the D-loop, anticodon loop, and TΨC-loop had high conservation scores (average conservation scores: S4, 7.76; S8, 8.38; and S12, 8.43). When the stem regions of the tRNA molecules were considered, the D-stem regions had high conservation scores (average conservation scores: S3, 8.01; S5, 8.09), whereas the conservation scores were relatively low in the other stem regions, compared with those in the loop regions (average conservation scores: S1, 5.69; S7, 5.07; S9, 5.10; S11, 6.26; S13, 6.17; S14, 5.55). We assume that the base pairs forming these stem regions have important functions, although the sequences are relatively variable (see below). The highly conserved regions include most of the interacting tRNA sequence regions mentioned in Figure 4 (S3, S8, and S15).

When we precisely compared the patterns of conserved tRNA positions among the three domains of life, the conservation patterns of each ribonucleotide were very similar in the Bacteria and Archaea. However it has been reported that a phylogenetic analysis of the aaRS sequences revealed a strong distinction between the bacterial and archaeal aaRSs (Woese et al., 2000). When we compared the conservation patterns between the prokaryotes and eukaryotes, the degree of conservation was relatively low. The conservation scores in the loop regions (S4, S8, and S12) were also high within the eukaryotes (average conservation scores: S4, 7.54; S8, 8.36; S12, 7.84), but in the anticodon (S7 and S9) and TΨC stem (S11 and S13) sequence regions, the level of conservation was slightly higher in eukaryotes (average conservation scores: S7, 5.72; S9, 5.5; S11, 6.59; S13, 6.54). It should be noted that the conservation of the first two base pairs (positions 1 and 72, and positions 2 and 71) in the acceptor stem (S1 and S14) region was markedly reduced in the eukaryotes (see Supplementary Figure S7A). Furthermore, over 59% (66,969 of 112,862) of the eukaryotic tRNAs did not conform to the conventional tRNA numbering rules shown in Figure 3A; for example, some eukaryotic tRNAs included an unusual bulge loop or had a shorter/longer stem. The numbers of conventional and non-conventional tRNAs observed in the three domains of life are summarized in Table 2. In fact, our research group has reported the presence of noncanonical tRNA rules in several eukaryotic tRNAs (Hamashima et al., 2016).

**Figure 6:**
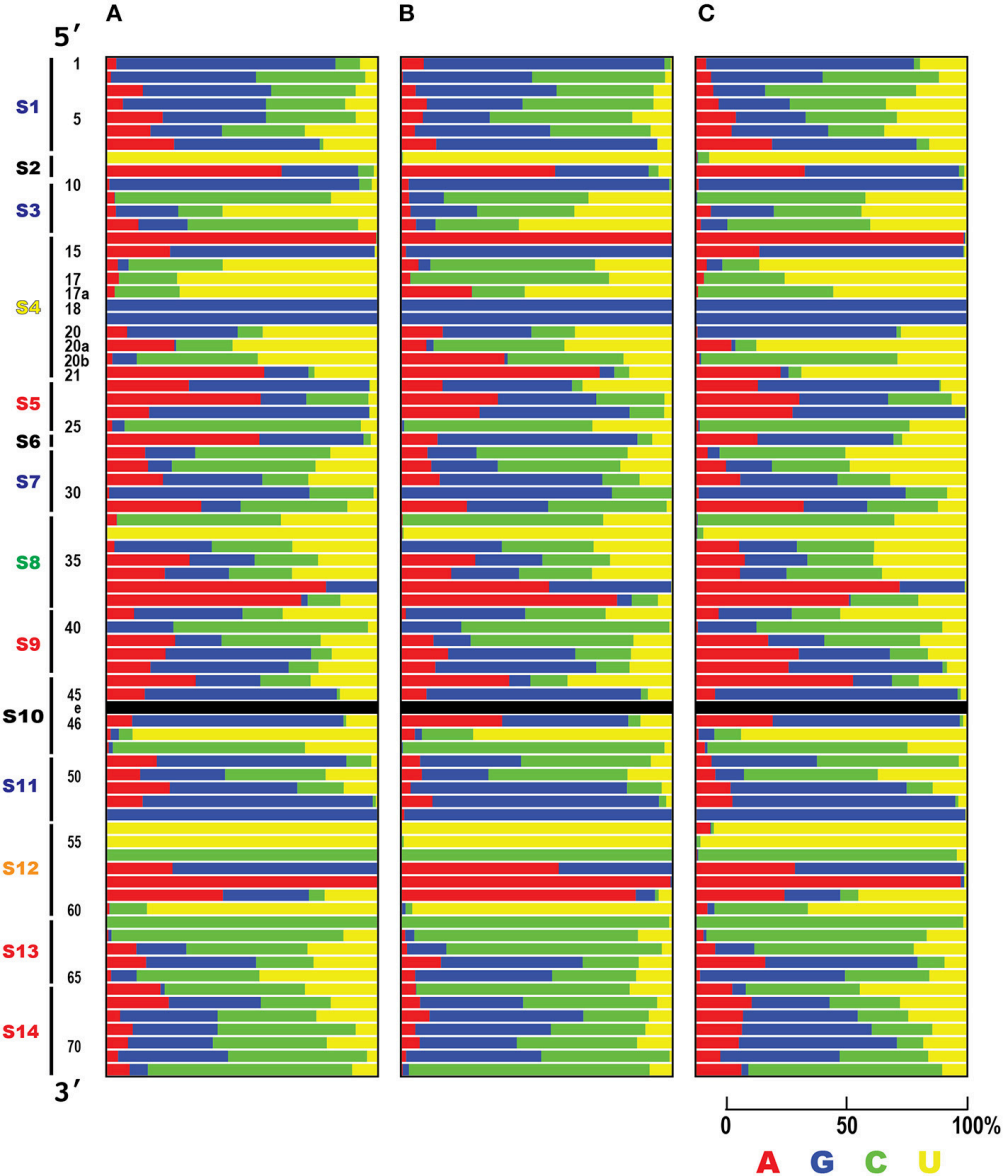
Summary of the nucleotide frequencies in tRNAs. Nucleotide frequencies for each tRNA position among the three domains of life, **(A)** Bacteria (83 species), **(B)** Archaea (182 species), and **(C)** Eukarya (150 species), are shown. The Y column represents the tRNA ribonucleotide positions based on the universal conventional tRNA positions (Sprinzl et al., 1998). Color indicates the abundance ratio (%) of each ribonucleotide (A, red; C, green; U, yellow; G, blue).

Is this conservation of tRNA ribonucleotides reflected by the base sequence specificity? To answer this question, the nucleotide frequency of each tRNA position was calculated for all 20 types of tRNAs (Figure 6). In all three domains of life, positions 8, 10, 14, 18, 19, 33, 53, 54, 55, 56, 58 and 61 are conserved or semi-conserved (Supplementary Figure S7B). Moreover, tRNA ribonucleotide positions are specifically conserved for the corresponding types of amino acids (Supplementary Figure S7A). Among these positions, two bases present in the anticodon (positions 35 and 36) are typical examples of bases conserved for specific cognate amino acids. The ribonucleotide pair (positions 1 and 72) next to the discriminator (position 73) is conserved for specific cognate amino acids, in that it is highly conserved in Bacteria but less conserved in Eukarya. The second ribonucleotide pair at the acceptor stem (positions 2 and 71) is also conserved in Archaea (Supplementary Figure S7A).

**Figure 7:**
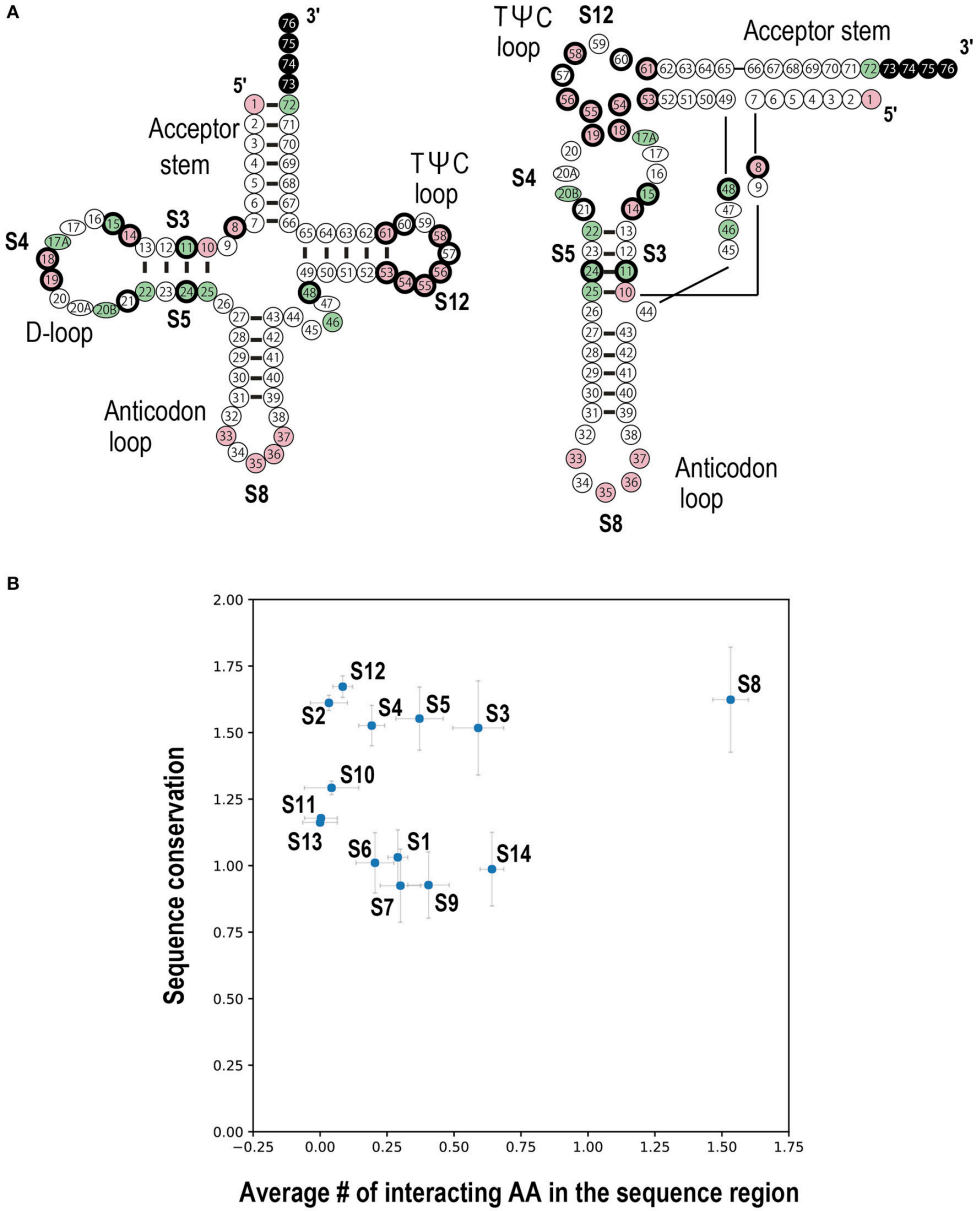
Summary of tRNA ribonucleotide conservation scores and interactions with the corresponding aaRSs in Bacteria. **(A)** The cloverleaf (left) and L-shaped (right) structures of conserved tRNA ribonucleotides. Positions shown in pink and green represent the conserved and semi-conserved ribonucleotides, respectively (see Supplementary Figure S7A). Ribonucleotides in the terminal CCA region are colored in black, because no conservation analysis was performed in this region. The circles with thick black edges represent the tRNA positions important for either L-shape or TΨC-loop formation (Giegé et al., 2012). This figure was adapted from Helm et al. (2000) with modifications. **(B)** Scatter plot of the number of interacting amino acids versus the sequence conservation score among tRNA sequence regions. See Figure 3A for the tRNA sequences (S1–S14). The *x*-axis indicates the average number of amino acids (AA) interacting with each sequence region. The *y*-axis indicates the average conservation score for the different sequence regions (see Figure 5). Error bars represent the standard error of the mean.

To summarize the relationships between the interacting ribonucleotides and conserved ribonucleotides, a schematic representation of the important ribonucleotides in the structures of select bacterial tRNAs and a scatter plot are presented (Figure 7). The anticodon loop (S8) and D-stem (S3 and S5) regions interact and have been conserved throughout bacterial evolution (Figure 7B). Because the terminal CCA is sometimes missing from the genomic sequence but added post-transcriptionally to the 3′ end of the corresponding tRNA, we could not estimate the degree of conservation in this region, although the CCA sequence is apparently highly conserved. Moreover, we found that two loop regions (D-loop (S4) and TΨC-loop (S12)) have also been conserved throughout bacterial evolution. It has been reported that tRNA ribonucleotides at positions 8, 11, 14, 15, 18, 19, 21, 24, 48, 55, and 56 are important for L-shape formation, while those at positions 53, 54, 57, 58, 60, and 61 are important for TΨC-loop formation (Giegé et al., 2012) (Figure 7A). These ribonucleotides are found mainly in the D-stem, D-loop, and TΨC-loop, and many are consistent with the conserved ribonucleotides identified in the current analysis. Note that the gap region (S2) between the accepter and D-stem is highly conserved, although there are only two ribonucleotides in this region; the position 8 ribonucleotides in this region are highly conserved and are important for L-shape formation. These observations are basically true in archaeal and eukaryotic tRNAs as well.

One factor that must also be considered together with the conservation of the tRNA sequence is the modification of specific tRNA ribonucleotides. Although not all the modified positions of tRNAs are known, *E. coli* tRNAs are reported to be modified at 19 positions: 8, 13, 16, 17, 18, 20, 20a, 32, 34, 37, 38, 39, 40, 46, 47, 54, 55, 65, and 67 (Björk and Hagervall, 2014). The positions of the modified sites are biased toward loop regions, in that 13 modified sites are located in loop regions, four in stem regions, and two in the V-arm region. Five of the 19 modified sites have high sequence conservation scores (positions 8, 18, 37, 54, and 55). Except for position 55, the positions corresponding to dihydrouridine (16, 17, 20, and 20a) and pseudouridine (Ψ-uridine) modifications (13, 38, 39, 40, 55, and 67) have relatively low conservation scores. The sequence ratios shown in Figure 6 indicate that these low-scoring positions do not always encode uridine. Interactions with the aaRSs were observed at six of the 19 reported modified positions (position 8, 13,16, 20, 34, and 37) in some complexes (Supplementary Figure S4). Meanwhile, we obtained information for each ribonucleotide modification from 21 PDB entries (Supplementary Table S1). We found that each artificial ribonucleotide modification within the 3′ end of bacterial tRNA^Leu^ (PDB IDs: 3zju, 4ari, 4as1, 3zjt, and 3zjv) enhanced the interaction between each ribonucleotide and aaRS (Supplementary Figure S4). However, because there were insufficient structural data for comparisons of each ribonucleotide with versus without modifications, we could not demonstrate the exact effects of other ribonucleotide modifications on the interaction. Based on these observations, we suggest that the modified positions are not always involved in the tRNA–aaRS interaction, but in some cases, they may contribute to the interaction. This should be clarified in future research.

## Concluding remarks and future prospects

In this study, we quantitatively analyzed the molecules involved in the interactive surface between tRNA and aaRS. A comparative analysis of tRNA–aaRS complexes was performed by mapping the interacting ribonucleotides in two-dimensional space, using the coordinates of the universal tRNA positioning rules and the specific regions of the cloverleaf tRNA structure. We successfully identified the interacting regions in the tRNA–aaRS complex and the evolutionarily conserved ribonucleotides in the tRNA molecule. Ribosomal proteins, elongation factors, as well as many tRNA-modifying enzymes are additional tRNA-binding partners (Kanai, 2014), and systematic analyses of the interacting surfaces of tRNA–aaRS complexes will open new doors in the study of tRNA evolution.

We emphasize again that we have developed a basic method for considering the relationships of the interacting molecules in the tRNA–aaRS complex. When the three-dimensional structures of DNA/RNA-protein complexes are available, our newly developed approach could be applied to these complexes. Therefore, the interacting regions between the components of these complexes can be visualized and the sequence conservation discussed. Here, our sequence conservation analysis identified many of the putative functional regions, and some of these regions may correspond to interacting regions. It is assumed that the DNA/RNA-binding regions of DNA/RNA-binding proteins are more strongly conserved than other regulatory regions. In other words, these DNA/RNA-binding regions may be evolutionarily fundamental. Therefore, using our approach, we can identify two sets of regional information: (a) the original and fundamental functional regions; and (b) the more recently acquired functional regions. For example, although the basic function of aaRSs is in the activation of amino acids and their transfer to specific RNAs, the enzymes of this group participate in other cell processes (Lee et al., 2004; Guo and Schimmel, 2013; Motzik et al., 2013). The approach developed here may allow the distinction of these two functional domains. We believe that this approach is also applicable to other complexes, such as transcription factors and their target DNAs, and long noncoding RNAs and their binding proteins. The methods demonstrated in this paper can also be applied to other complexes, including translation initiation complexes, spliceosomes, and ribosomes.

## Author contributions

ST and AK conceived and designed the study, and wrote the manuscript. ST and HS performed the analyses. ST, MT, and AK edited the manuscript. All authors have read and approved the final manuscript.

### Conflict of interest statement

The authors declare that the research was conducted in the absence of any commercial or financial relationships that could be construed as a potential conflict of interest.
