# Supplementary material for: Systematic Analysis of the Binding Surfaces between tRNAs and Their Respective Aminoacyl tRNA Synthetase Based on Structural and Evolutionary Data
Source: Front Genet. 2018 Jan 8;8:227. doi: 10.3389/fgene.2017.00227 (PMC5766645; doi:10.3389/fgene.2017.00227)
Supplement: Supplementary file 3 [file Image1.PDF]

Supplementary Material

Systematic analysis of the binding surfaces between tRNAs and their respective aminoacyl tRNA synthetase based on structural and evolutionary data

Satoshi Tamaki[1,2], Masaru Tomita[1,2,3], Haruo Suzuki[1,3] and Akio Kanai[1,2,3,*]

[1]Institute for Advanced Biosciences, Keio University, Tsuruoka 997-0017, Japan

[2]Systems Biology Program, Graduate School of Media and Governance, Keio University, Fujisawa 252-0882, Japan

[3]Faculty of Environment and Information Studies, Keio University, Fujisawa 252-0882, Japan

*** Correspondence:** Akio Kanai email: akio@sfc.keio.ac.jp

Supplementary Table S1. Summary of tRNAs and their aminoacyl-tRNA synthetases (aaRSs) used in this study.

(A) Bacteria

tRNA type (anticodon)	tRNA length (nt) §1	aaRS length (aa) §2	aaRS class	PDB ID	Resolution (Å)	Species (tRNA / aaRS) §3	Modified ribonucleotide (tRNA position : modification§4)
Cys (GCA)	74/74/74	461/461	Ia	1U0B	2.3	*E. coli*	
Ile (GAT)	75/75/n.d.	917/917	Ia	1FFY	2.2	*S. aureus*	
Leu (TAA)	71/87/87	813/880	Ia	4ARC	2.0	*E. coli*	
Leu (TAA)	80/87/87	820/880	Ia	3ZJU	2.4	*E. coli*	76:DJF
Leu (TAA)	80/87/87	821/880	Ia	4ARI	2.08	*E. coli*	76:N79
Leu (TAA)	79/87/87	860/880	Ia	4AQ7	2.5	*E. coli*	
Leu (TAA)	82/88/87	860/880	Ia	3ZGZ	2.4	*E. coli*	
Leu (TAA)	83/87/87	812/880	Ia	4AS1	2.02	*E. coli*	76:N79
Leu (TAA)	83/88/87	820/880	Ia	3ZJT	2.2	*E. coli*	76:574
Leu (TAA)	85/87/87	813/880	Ia	3ZJV	2.31	*E. coli*	76:365
Leu (n.d.)	82/82/87	860/880	Ia	4CQN	2.5	*E. coli*	
Met (CAT)	74/74/77	465/497	Ia	2CT8	2.7	*A. aeolicus*	
Met (CAT)	75/75/77	464/497	Ia	2CSX	2.7	*A. aeolicus*	
Val (CAC)	75/75/75	862/862	Ia	1GAX	2.9	*T. thermophilus*	
Val (CAC)	75/75/75	862/862	Ia	1IVS	2.9	*T. thermophilus*	
Gln (CTG)	71/75/75	536/553	Ib	4JXX	2.3	*E. coli*	
Gln (TTG)	71/75/75	538/553	Ib	4JXZ	2.4	*E. coli*	
Gln (CTG)	73/73/75	529/548	Ib	1EXD	2.7	*E. coli*	
Gln (CTG)	73/74/75	529/548	Ib	1EUY	2.6	*E. coli*	
Gln (CTG)	74/74/75	529/553	Ib	1GTR	2.5	*E. coli*	
Gln (CTG)	74/74/75	529/553	Ib	1GTS	2.8	*E. coli*	
Gln (CTG)	74/75/75	529/553	Ib	1QRS	2.6	*E. coli*	
Gln (CTG)	74/75/75	529/553	Ib	1QRT	2.7	*E. coli*	
Gln (CTG)	74/75/75	529/553	Ib	1QRU	3.0	*E. coli*	
Gln (CTG)	74/75/75	529/553	Ib	1QTQ	2.25	*E. coli*	
Gln (CTG)	74/75/75	529/556	Ib	2RD2	2.6	*E. coli*	
Gln (CTG)	74/75/75	529/556	Ib	2RE8	2.6	*E. coli*	
Gln (CTG)	74/75/75	529/553	Ib	1ZJW	2.5	*E. coli*	
Gln (CTG)	74/75/75	529/554	Ib	1O0B	2.7	*E. coli*	
Gln (CTG)	74/75/75	529/554	Ib	1O0C	2.7	*E. coli*	
Glu (CTG)	74/74/74	463/487	Ib	3AKZ	2.9	*T. martima*	
Glu (CTC)	74/75/75	468/468	Ib	1N77	2.4	*T. thermophilus*	
Glu (CTC)	75/75/75	468/468	Ib	1N78	2.1	*T. thermophilus*	
Glu (CTC)	75/75/75	468/468	Ib	2CV1	2.41	*T. thermophilus*	
Glu (CTC)	75/75/75	468/468	Ib	2CV2	2.69	*T. thermophilus*	
Glu (CTC)	75/75/75	468/468	Ib	2DXI	2.2	*T. thermophilus*	
Glu (CTC)	75/75/75	468/468	Ib	2CV0	2.4	*T. thermophilus*	
Glu (CTC)	75/75/75	468/468	Ib	1G59	2.4	*T. thermophilus*	

Tyr (GTA)	84/86/86	(427/432) ×2	Ic	1H3E	2.9	*T. thermophilus*	35:PSU , 54:5MU , 55:PSU , 58:1MA
His (GTG)	77/78/77	409/423	IIa	4RDX	2.55	*T. thermophilus*	-1:GTP
Pro (CGG)	67/77/77	465/477	IIa	1H4Q	3.0	*T. thermophilus*	54:5MU , 55:PSU
Ser (GGA)	65/94/94	372/421	IIa	1SER	2.9	*T. thermophilus*	20a:H2U , 54:5MU , 55:PSU
Thr (CGT)	76/76/76	641/642	IIa	1QF6	2.9	*E. coli*	16:H2U , 17:H2U , 20:H2U , 37:AET , 46:G7M , 54:5MU , 55:PSU
Asp (GTC)	73/73/n.d.	580/580	IIb	1EFW	3.0	*T. thermophilus*	8:4SU , 16:H2U , 20:H2U , 20a:H2U , 34:QUO , 37:2MA , 46:G7M , 54:5MU , 55:PSU , 65:PSU ,
Asp (GTC)	75/75/n.d.	585/590	IIb	1IL2	2.6	*S.cerevisiae / E. coli*	13:PSU , 16:H2U , 19:H2U , 32:PSU , 37:1MG , 49:5MC , 54:5MU , 55:PSU ,
Asp (GTC)	77/77/77	585/585	IIb	1C0A	2.4	*E. coli*	8:4SU , 16:H2U , 20:H2U , 20a:H2U , 34:QUO , 46:G7M , 54:5MU , 55:PSU , 65:PSU
Phe (GAA)	76/76/76	(336/350) ×2 (alpha) (781/785) ×2 (beta)	IIc	2IY5	3.1	*T. thermophilus*	
Phe (GAA)	76/76/76	(345/350) ×2 (alpha) (785/785) ×2 (beta)	IIc	1EIY	3.3	*T. thermophilus*	

(B) Archaea

tRNA type (anticodon)	tRNA length (nt) §1	aaRS length (aa) §2	aaRS class	PDB ID	Resolution (Å)	Species	Modified ribonucleotides (tRNA position : modification)§3
Leu (CAA)	88/88/85	948/967	Ia	1WZ2	3.21	*P. horikoshii*	
Tyr (GTA)	75/77/77	(299/306) ×2	Ic	1J1U	1.95	*M. jannaschii*	

(C) Eukarya

tRNA type (anticodon)	tRNA length (nt) §1	aaRS length (aa) §2	aaRS class	PDB ID	Resolution (Å)	Species (tRNA / aaRS) §4	Modified ribonucleotides (tRNA position : modification)§3
Arg (ICG)	75/76/73	606/607	Ia	1F7U	2.2	*S.cerevisiae*	1:PSU , 9:1MG , 10:2MG , 16:H2U , 19:H2U , 26:M2G ,

							27:PSU , 47:H2U , 49:5MC , 54:5MU , 55:PSU , 58:1MA
Arg (ICG)	72/76/73	606/607	Ia	1F7V	2.9	*S.cerevisiae*	1:PSU , 9:1MG , 10:2MG , 16:H2U , 19:H2U , 26:M2G , 27:PSU , 47:H2U , 49:5MC , 54:5MU , 55:PSU , 58:1MA
Trp (CCA)	72/72/72	(373/471) ×2	Ic	2AKE	3.1	*B.taurus / H.sapiens*	
Trp (CCA)	75/75/72	(373/471) ×2	Ic	2DR2	3.0	*B.taurus / H.sapiens*	
Gly (CCC)	69/74/71	(458/739) ×2	IIa	4KR2	3.29	*H.sapiens*	1:GTP
Gly (CCC)	70/74/71	(466/739) ×2	IIa	4KR3	3.23	*H.sapiens*	1:GTP
Gly (CCC)	74/74/71	(519/739) ×2	IIa	5E6M	2.93	*H.sapiens*	1:GTP
Gly (CCC)	69/69/71	(562/739) ×2	IIa	4QEI	2.88	*H.sapiens*	1:GTP
Asp (GTC)	75/75/72	490/557	IIb	1ASY	2.9	*S.cerevisiae*	13:PSU , 16:H2U , 19:H2U , 32:PSU , 37:1MG , 49:5MC , 54:5MU , 55:PSU
Asp (GTC)	75/75/72	490/557	IIb	1ASZ	3.0	*S.cerevisiae*	13:PSU , 16:H2U , 19:H2U , 32:PSU , 37:1MG , 49:5MC , 54:5MU , 55:PSU

n.d.: not determined.

×2: protein dimer.

§1 Nucleotide (nt) length of tRNA structurally determined / tRNA length used for the structural determination experiment / tRNA length in the original species.

§2 Structurally determined amino acid (aa) length of aaRS / aaRS length used in the structural analysis.

§3 If two species names occur in one column, the first is the organism from which the tRNA was taken and the second is the organism from which the aaRS was taken.

§4 Abbreviations:

1MA	6-hydro-1-methyladenosine-5'-monophosphate
1MG	1N-methylguanosine-5'-monophosphate
2MA	2-methyladenosine-5'-monophosphate
2MG	2N-methylguanosine-5'-monophosphate
365	[(1S,5R,6R,7'S,8R)-7'-(aminomethyl)-6-(6-aminopurin-9-yl)-2'-(3-oxidanylpropoxy)spiro[2,4,7-trioxa-3-boranuidabicyclo[3.3.0]octane-3,9'-8-oxa-9-boranuidabicyclo[4.3.0]nona-1(6),2,4-triene]-8-yl]methyl dihydrogen phosphate
4SU	4-thiouridine-5'-monophosphate
574	[(3aS,4R,6R,6aR)-2-{2-[(1S)-2-amino-1-hydroxyethyl]phenyl}-6-(6-amino-9H-purin-9-yl)tetrahydrofuro[3,4-d][1,3,2]dioxaborol-4-yl]methyl dihydrogen phosphite

5MC	5-methylcytidine-5'-monophosphate
5MU	5-methyluridine 5'-monophosphate
AET	N-[N-(9-B-D-ribofuranosylpurin-6-yl)methylcarbamoyl]threonine-5'-monophosphate
DJF	[(1S,5R,6R,8R)-6-(6-aminopurin-9-yl)-2'-(3-oxidanylpropoxy)spiro[2,4,7-trioxa-3-boranuidabicyclo[3.3.0]octane-3,9'-8-oxa-9-boranuidabicyclo[4.3.0]nona-1(6),2,4-triene]-8-yl]methyl dihydrogen phosphate
G7M	N7-methyl-guanosine-5'-monophosphate
GTP	guanosine-5'-triphosphate
H2U	5,6-dihydrouridine-5'-monophosphate
M2G	N2-dimethylguanosine-5'-monophosphate
N79	[(1S,5R,6R,8R)-6-(6-aminopurin-9-yl)spiro[2,4,7-trioxa-3-boranuidabicyclo[3.3.0]octane-3,9'-8-oxa-9-boranuidabicyclo[4.3.0]nona-1(6),2,4-triene]-8-yl]methyl dihydrogen phosphate
PSU	pseudouridine-5'-monophosphate
QUO	2-amino-7-deaza-(2'',3''-dihydroxy-cyclopentylamino)-guanosine-5'-monophosphate

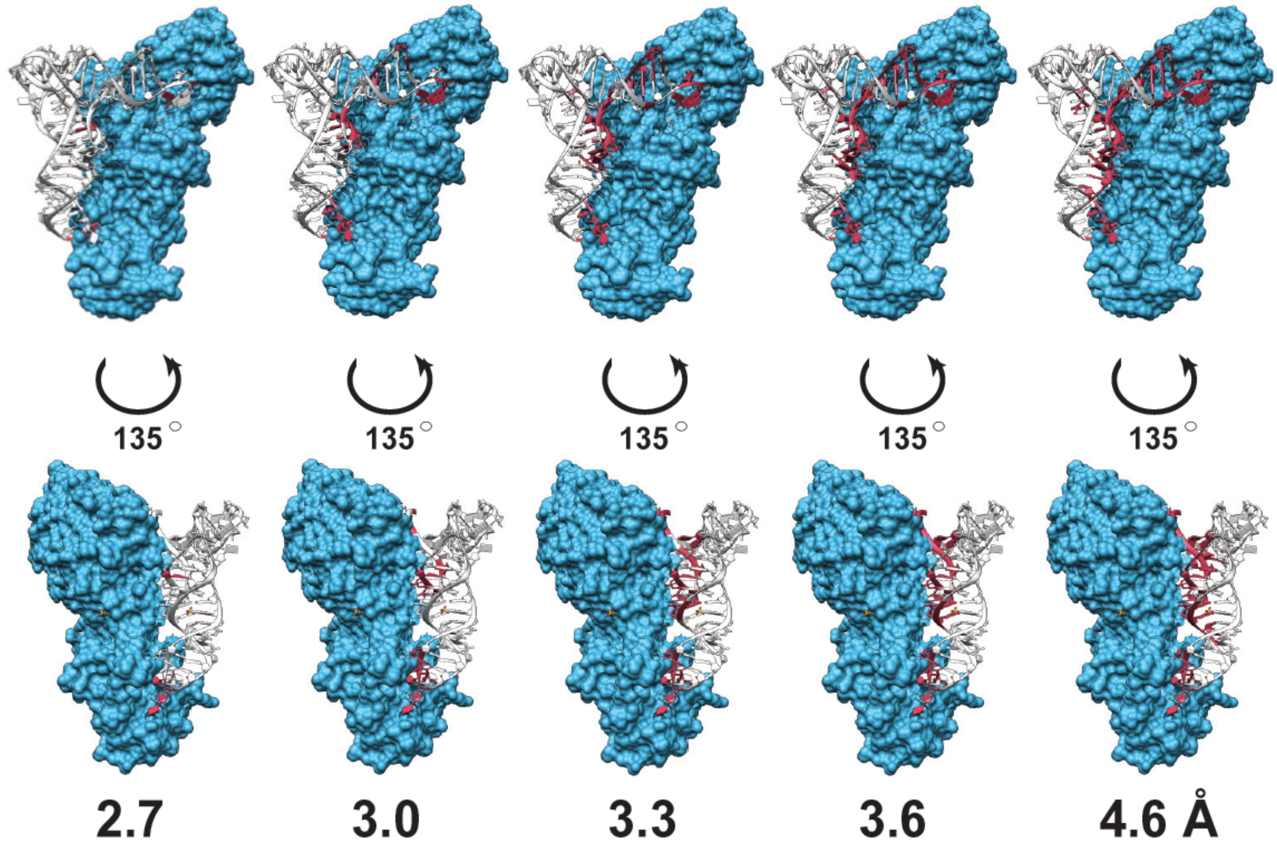

Supplementary Figure S1. Five examples of $tRNA^{Gln}$ ribonucleotides at various distances from GlnRS. Three-dimensional structure of the $tRNA^{Gln}$–GlnRS complex (PBD ID: 1exd), shown in Figure IB, is used as an example. Nucleotides at different distances (2.7, 3.0, 3.3, 3.6, or 4.6 Å) from GlnRS are colored red. 3.3 Å was used as the interacting distance in this study. Top row and bottom row show the complex rotated 135°.

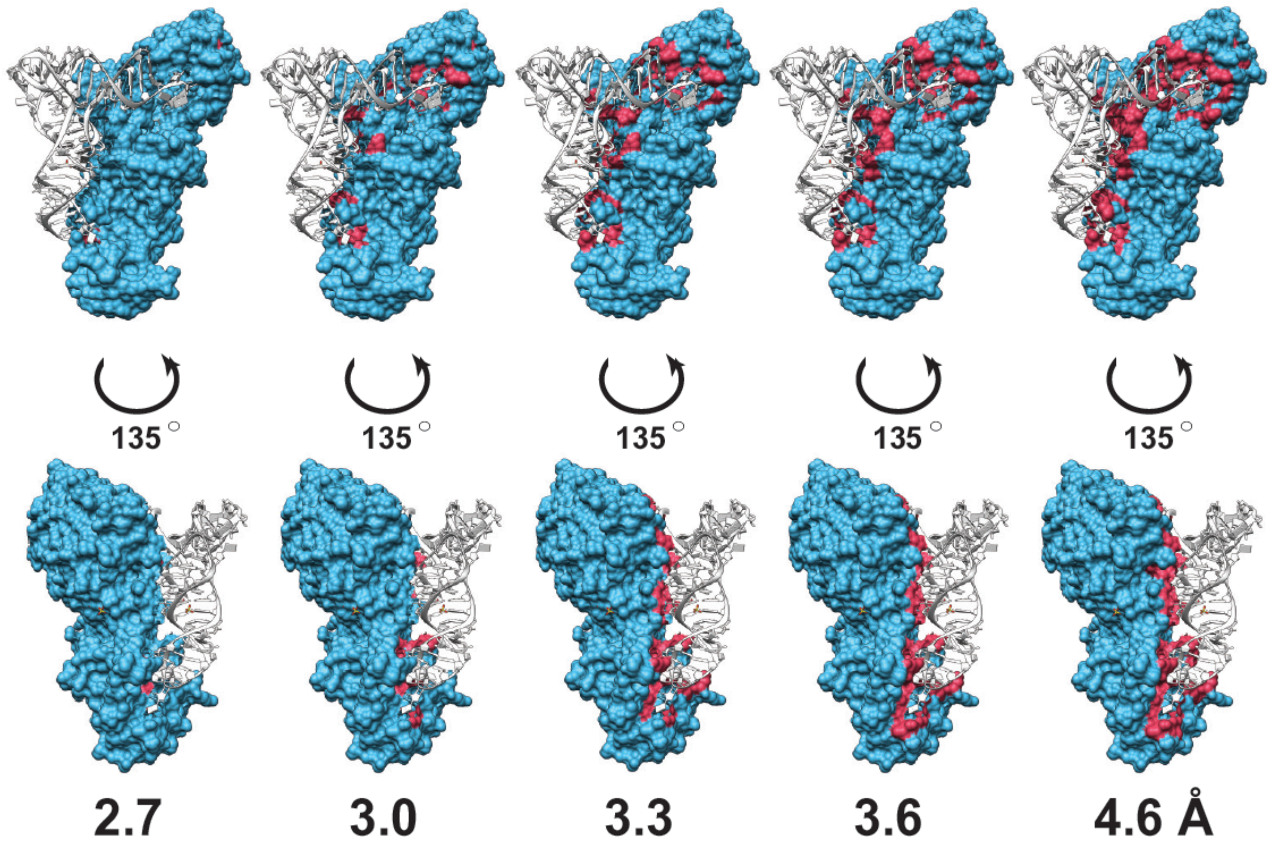

Supplementary Figure S2. Five examples of amino acid residues of GlnRS at various distances from tRNAGln. Three-dimensional structure of the tRNAGln –GlnRS complex (PBD ID: 1exd), shown in Figure IB, is used as an example. Amino acids at different distances (2.7, 3.0, 3.3, 3.6, or 4.6 Å) from tRNAGln are colored red. 3.3 Å was used as the interacting distance in this study. Top row and bottom row show the complex rotated 135°.

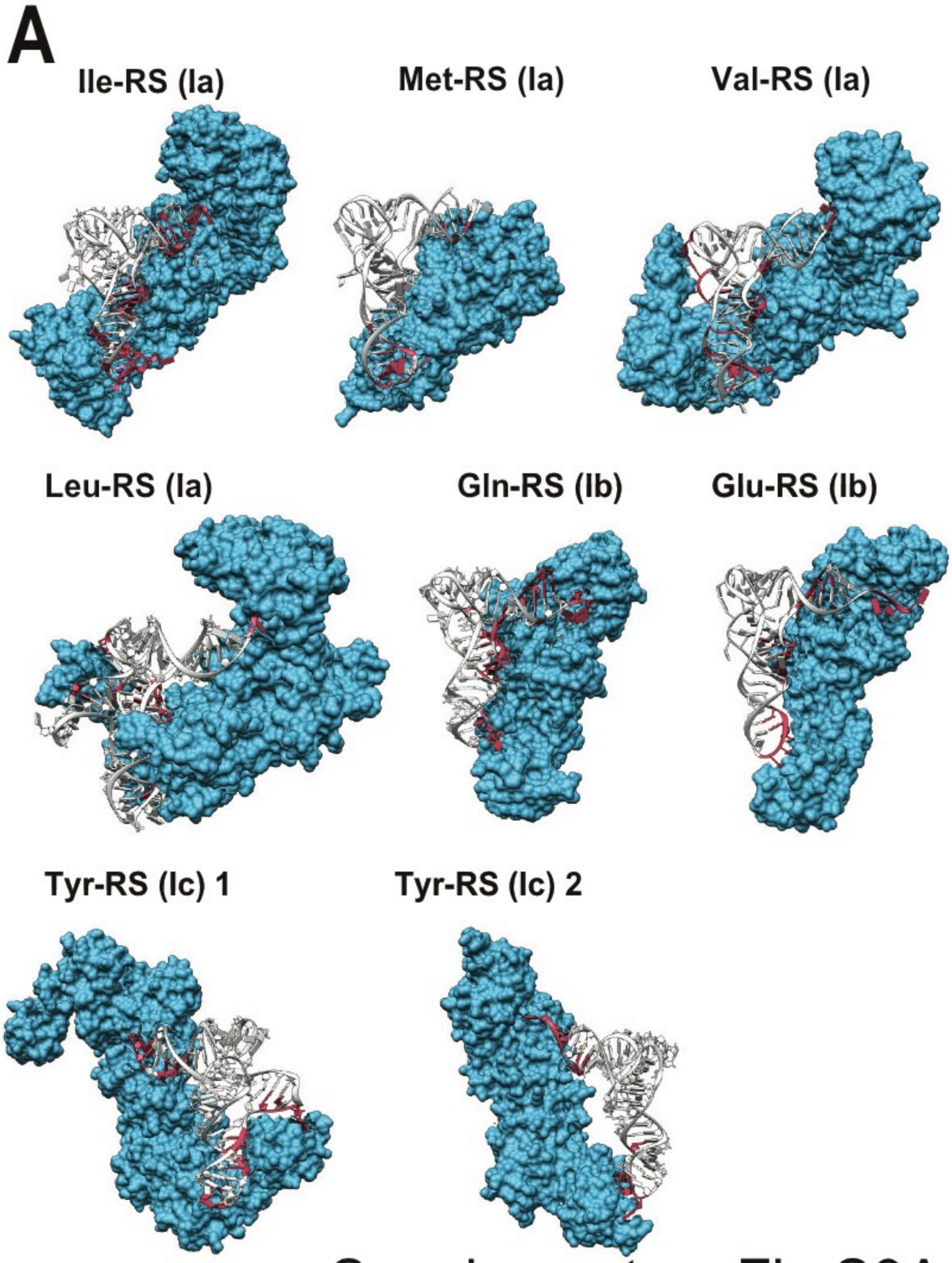

Supplementary Fig.S3A

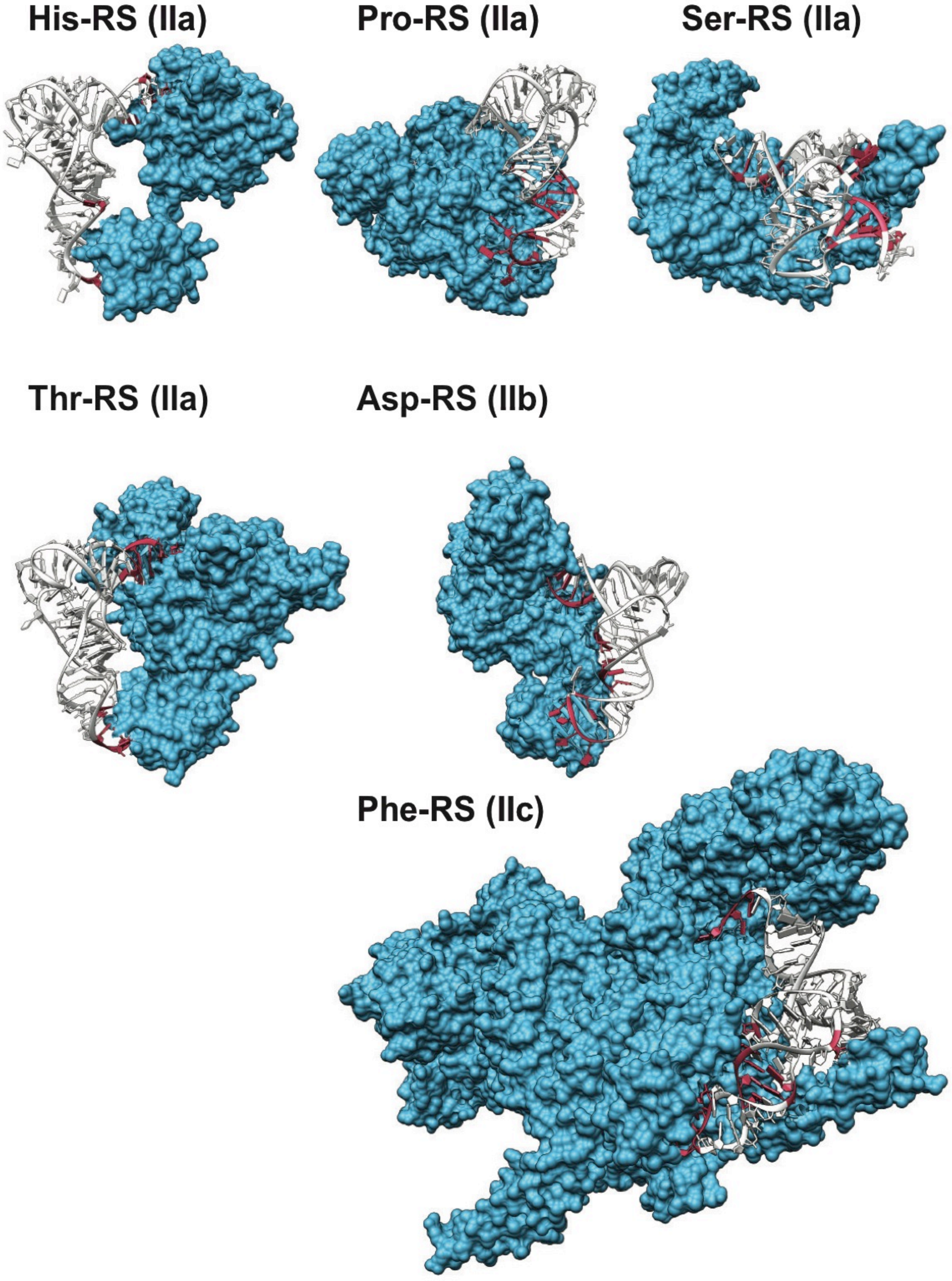

Supplementary Fig.S3A continued

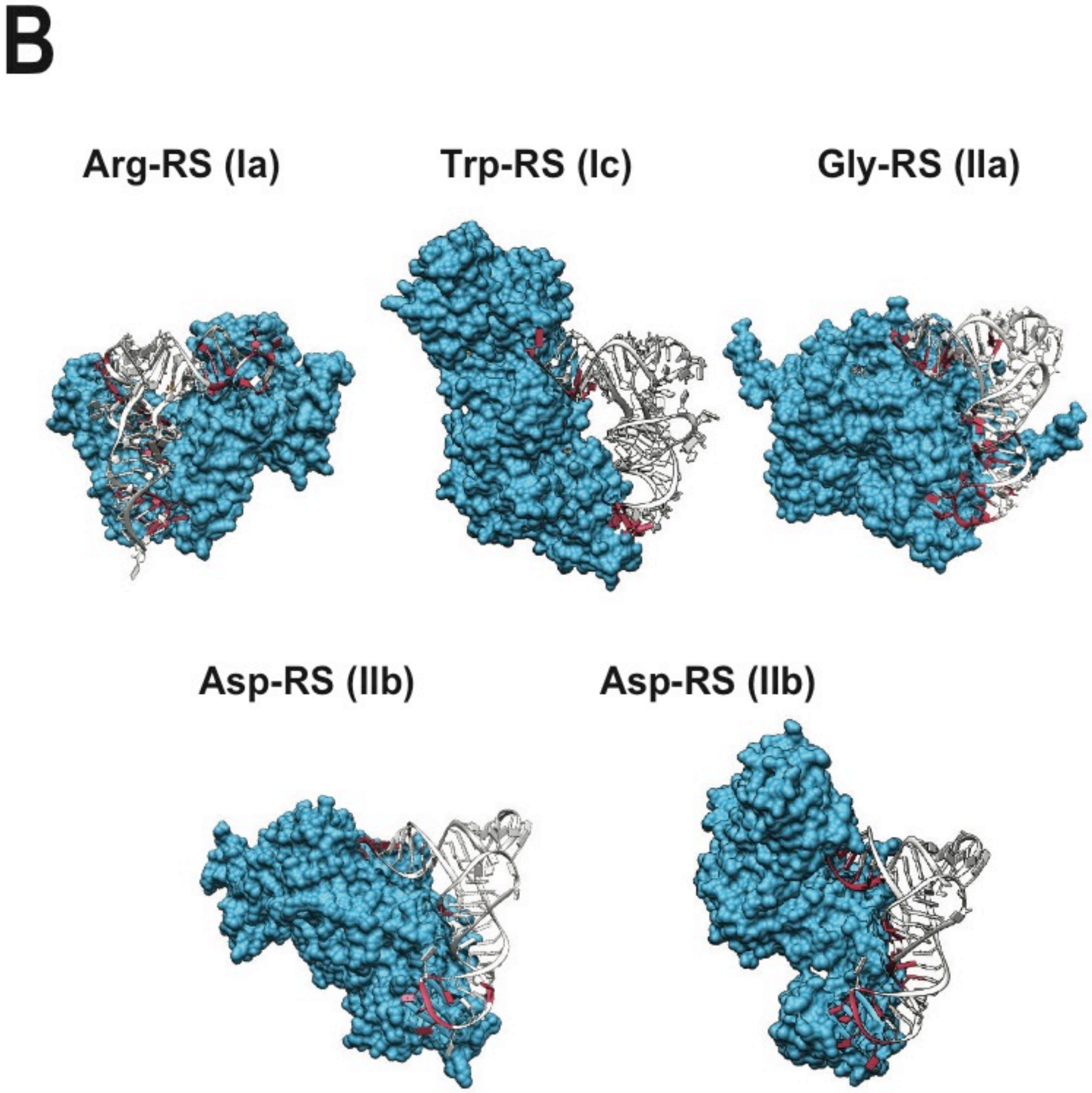

Supplementary Fig.S3B

Supplementary Figure S3. Various tRNA–aaRS complexes and their interacting ribonucleotides. Three-dimensional illustrations of aaRSs complexed with their corresponding tRNAs. Amino acid residues in each aaRS are colored blue; nucleotides in tRNA within a distance of 3.3 Å from the aaRS are colored red; and other nucleotides in tRNA are colored white. (A) Fourteen types of prokaryotic tRNA–aaRS complexes. RS class (Ia, Ib, Ic, IIa, IIb, or IIc) and aaRS type (PDB ID) are shown as follows: Ia: Ile (1ffy), Met (2csx), Val (1gax), Leu (3zjv); Ib: Gln (1exd), Glu (1g59); Ic: Tyr-1 (h3e), Tyr-2 (1j1u); IIa: His (4rdx), Pro (1h4q), Ser, (3w3s), Thr (1qf6); and IIb: Asp (1il2); IIc: Phe (1eiy). Note that the two TyrRS shown in this figure are homodimeric (α2) protein complexes (1h3e and 1j1u), and PheRS shown in this figure are tetrameric (α2β2) protein complexes (1eiy). (B) Illustrations of five types of eukaryotic tRNA–aaRS complexes. RS class (Ia, Ic, IIa, or IIb) and aaRS type (PDB ID) are shown as follows: Ia Arg (1f7u); Ic: Trp (2ake); IIa: Gly (4kr3); and IIb: Asp (1asy), Asp (1il2). Note that ArgRS (1f7u) and GlyRS (4kr3) are homodimeric (α2) protein complexes.

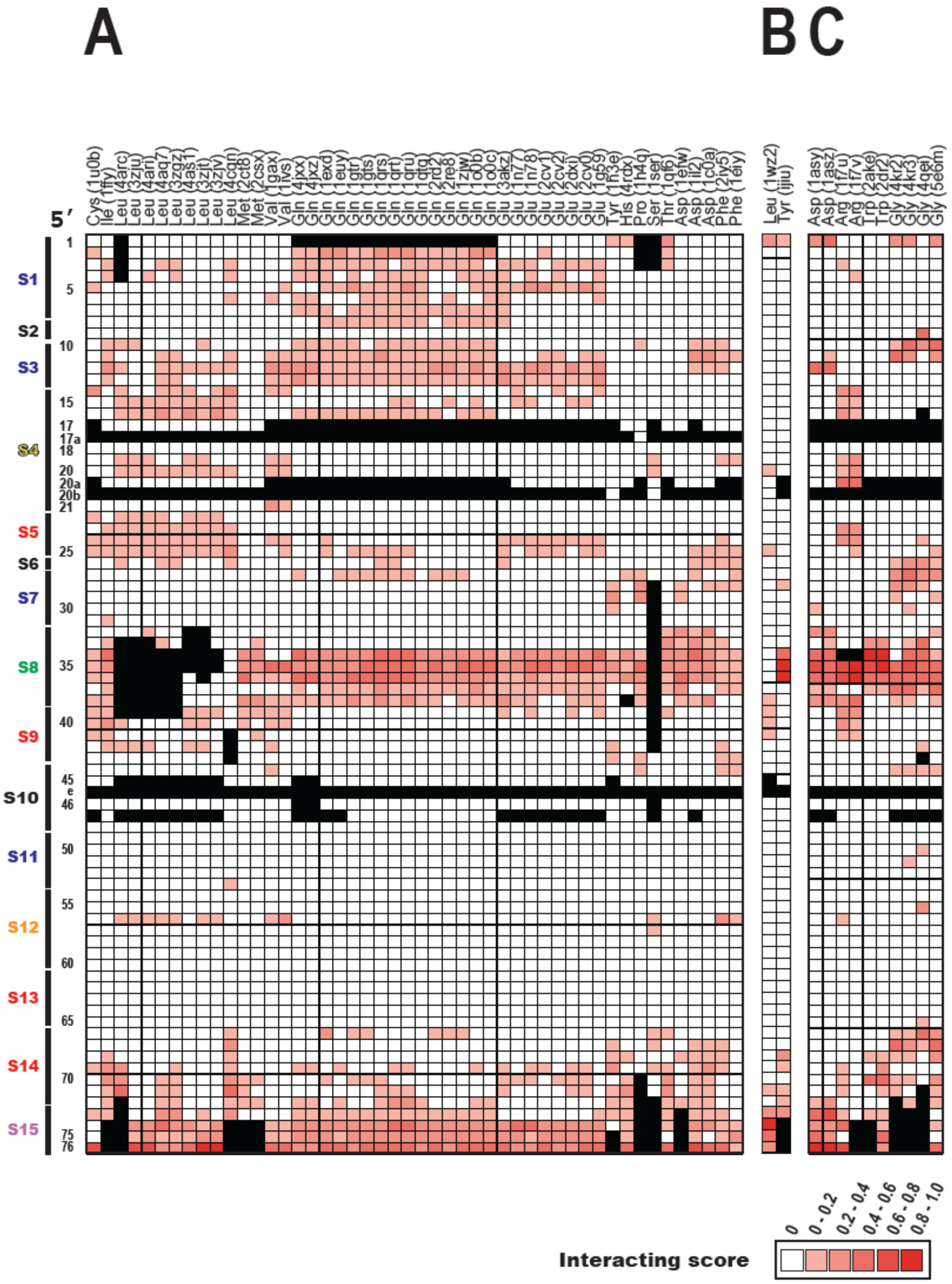
A
B C
5′
S1
S2
S3
S4
S5
S6
S7
S8
S9
S10
S11
S12
S13
S14
S15
Cys (1u0b)
Ile (1ffy)
Leu (4arc)
Leu (3zju)
Leu (4ari)
Leu (4aq7)
Leu (3zgz)
Leu (4as1)
Leu (3zjt)
Leu (3zjv)
Leu (4cqn)
Met (2ct8)
Met (2csx)
Val (1gax)
Val (1ivs)
Gln (4jxx)
Gln (4jxz)
Gln (1exd)
Gln (1euy)
Gln (1gtr)
Gln (1gts)
Gln (1qrs)
Gln (1qrt)
Gln (1qru)
Gln (1qtq)
Gln (2rd2)
Gln (2re8)
Gln (1zjw)
Gln (1o0b)
Gln (1o0c)
Glu (3akz)
Glu (1n77)
Glu (1n78)
Glu (2cv1)
Glu (2cv2)
Glu (2dxi)
Glu (2cv0)
Glu (1g59)
Tyr (1h3e)
His (4rdx)
Pro (1h4q)
Ser (1ser)
Thr (1qf6)
Asp (1efw)
Asp (1il2)
Asp (1c0a)
Phe (2iy5)
Phe (1eiy)
Leu (1wz2)
Tyr (1j1u)
Asp (1asy)
Asp (1asz)
Arg (1f7u)
Arg (1f7v)
Trp (2ake)
Trp (2dr2)
Gly (4kr2)
Gly (4kr3)
Gly (4qei)
Gly (5e6m)
Interacting score
0
0 - 0.2
0.2 - 0.4
0.4 - 0.6
0.6 - 0.8
0.8 - 1.0

Supplementary Figure S4. Summary of the interacting ribonucleotides in the tRNA–aaRS complexes at single-ribonucleotide resolution. The Y column on the left side of the figure represents the tRNA ribonucleotide positions based on the universal conventional tRNA positions (Ref S1), with the tRNA sequence regions (S1–S15) described in Figure 3. Heatmap of the interacting scores in (A) Bacteria, (B) Archaea, and (C) Eukarya is shown (see also Figure 4). Red colors in each cell indicate six ranks of the interacting scores. Note that the maximum interacting amino acid number (i.e., interacting score = 1.0) differs among the three domains: Bacteria, 11; Archaea, 6; Eukarya, 7. Missing ribonucleotides in the tRNAs, structurally undetermined ribonucleotides, and tRNA positions with prefix 'e' (part of V-arm) were excluded from the analysis and the cells are colored black.

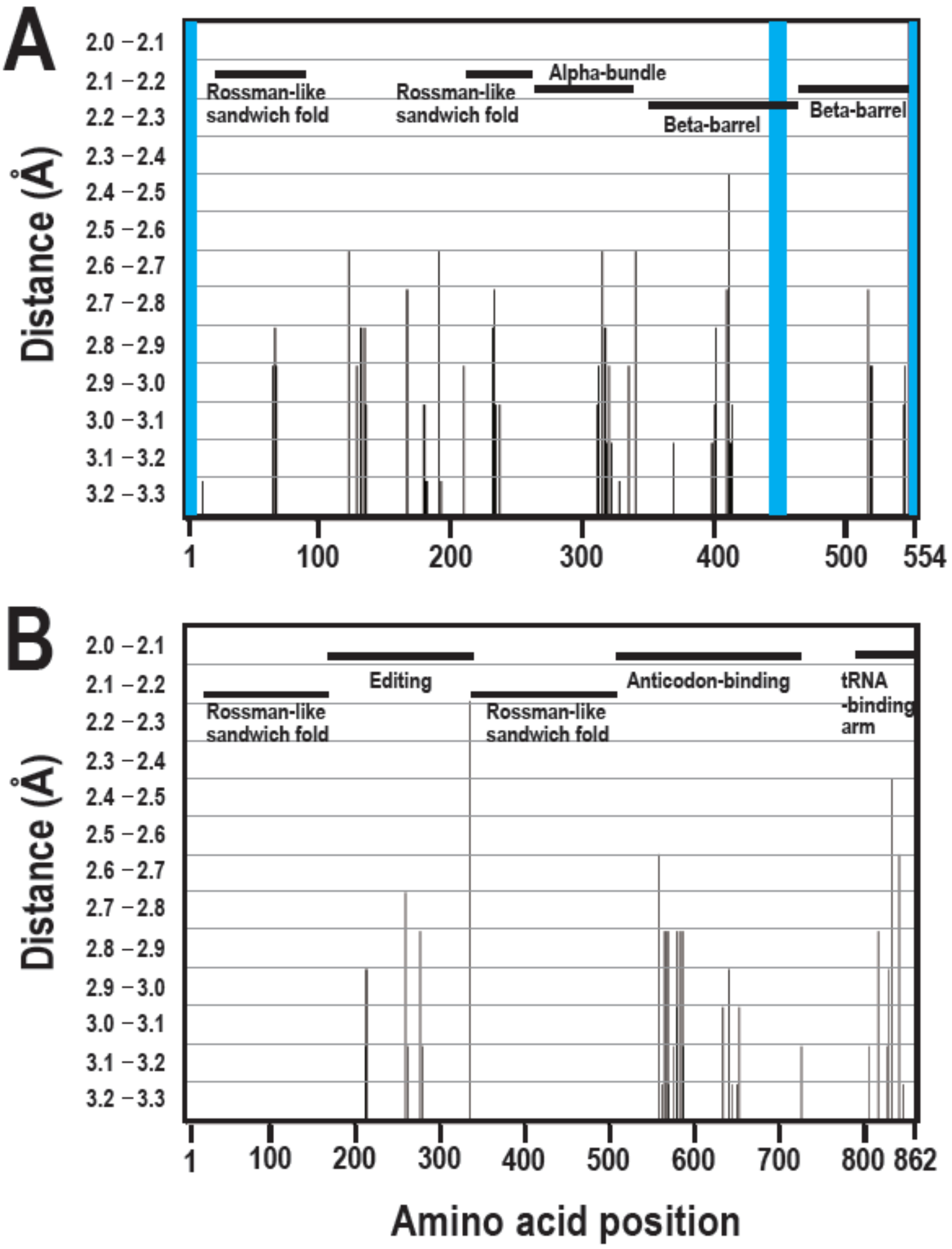
A
Distance (Å)
2.0 – 2.1
2.1 – 2.2
2.2 – 2.3
2.3 – 2.4
2.4 – 2.5
2.5 – 2.6
2.6 – 2.7
2.7 – 2.8
2.8 – 2.9
2.9 – 3.0
3.0 – 3.1
3.1 – 3.2
3.2 – 3.3
Rossman-like sandwich fold
Rossman-like sandwich fold
Alpha-bundle
Beta-barrel
Beta-barrel
1
100
200
300
400
500
554
B
Distance (Å)
2.0 – 2.1
2.1 – 2.2
2.2 – 2.3
2.3 – 2.4
2.4 – 2.5
2.5 – 2.6
2.6 – 2.7
2.7 – 2.8
2.8 – 2.9
2.9 – 3.0
3.0 – 3.1
3.1 – 3.2
3.2 – 3.3
Rossman-like sandwich fold
Editing
Rossman-like sandwich fold
Anticodon-binding
tRNA -binding arm
1
100
200
300
400
500
600
700
800
862
Amino acid position

Supplementary Figure S5. Mapping of the interacting amino acids in bacterial tRNA–aaRS complexes. Two-dimensional maps of the interacting amino acids in the tRNAGln–GlnRS complex (A) and the tRNAVal–ValRS complex (B). The x-axis indicates the amino acid positions of the aaRS, and the y-axis indicates the Euclidean distances between the tRNA ribonucleotides and the aaRS amino acids. Domain information was obtained from the InterPro database and Gene3D (Refs S2 and S3). Structurally undetermined amino acids and gapped regions, determined from the alignment of the corresponding sequences in InterPro, are shown in the vertical blue bar.

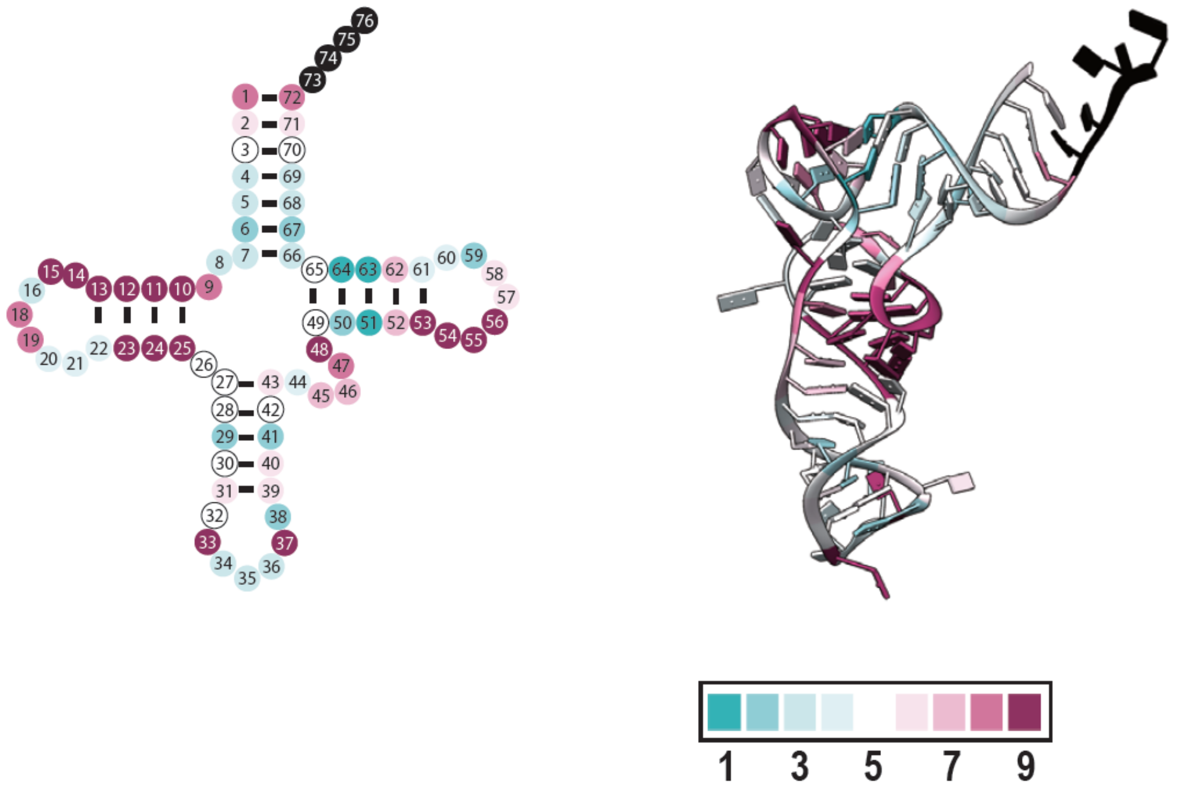

Supplementary Figure S6. Conservation scores mapped to the tRNA cloverleaf structure and L-shaped structure. Two- and three-dimensional structures of tRNA in the tRNAVal–ValRS complex (PBD ID: 1gax), shown in Figure IB, are used as examples to map conservation scores. Ribonucleotide conservation scores are represented as nine ranks, ranging from variable (rank 1: cyan) to conserved (rank 9: magenta). Numbers shown on the structures indicate the tRNA positions based on the universal tRNA positioning rules. The CCA terminal sequence region was not used in this analysis and colored black. The arrangement and color scheme used to map the conservation scores were according to ConSurf (Ref S4).

tRNA position	A: Bacteria	A: Archaea	A: Eukarya	B: Bacteria	B: Archaea	B: Eukarya
1	conserved	conserved	semi-conserved			
2		conserved				
3						
4						
5						
6						
7						
8	conserved	conserved	conserved	conserved	conserved	semi-conserved
9						
10	conserved	conserved	conserved	semi-conserved	conserved	conserved
11	semi-conserved		semi-conserved			
12						
13						
14	conserved	conserved	conserved	conserved	conserved	conserved
15	semi-conserved	conserved	semi-conserved		conserved	
16						
17						
17a	semi-conserved		conserved			
18	conserved	conserved	conserved	conserved	conserved	conserved
19	conserved	conserved	conserved	conserved	conserved	conserved
20						
20a						
20b	semi-conserved					
21						
22	semi-conserved					
23						
24	semi-conserved	semi-conserved	semi-conserved			
25	semi-conserved	semi-conserved	semi-conserved			
26						
27						
28						
29						
30		semi-conserved				
31						
32		semi-conserved	semi-conserved			
33	conserved	conserved	conserved	conserved	conserved	conserved
34						
35	conserved	conserved	conserved			
36	conserved	conserved	conserved			
37	conserved	semi-conserved	semi-conserved			
38						
39						
40		semi-conserved				
41						
42						
43						
44						
45						
e						
46	semi-conserved					
47						
48	semi-conserved	conserved	semi-conserved		conserved	
49						
50						
51						
52						
53	conserved	conserved	conserved	conserved	conserved	conserved
54	conserved	conserved	conserved	conserved	conserved	semi-conserved
55	conserved	conserved	conserved	conserved	conserved	conserved
56	conserved	conserved	conserved	conserved	conserved	conserved
57						
58	conserved	conserved	conserved	conserved	conserved	conserved
59						
60		conserved			conserved	
61	conserved	conserved	conserved	conserved	conserved	conserved
62						
63						
64						
65						
66						
67						
68						
69						
70						
71		conserved				
72	semi-conserved	conserved	semi-conserved			

Supplementary Figure S7. Summary of the conserved and semi-conserved tRNA positions in the three domains of life. (A) Amino-acid-specific conserved tRNA positions, based on the data used in Figure 5. Conservation scores were calculated from the mean entropy of the 20 amino acids. (B) Globally conserved tRNA ribonucleotide positions, based on the sequence frequencies shown in Figure 6. For both figures, conservation scores were calculated using Claude Shannon's entropy and divided into nine ranks. Conserved tRNA positions (rank 9: highest conservation score) are shown in pink, semi-conserved tRNA positions (rank 8) are shown in green, and all other regions are shown in black.

References:

S1. Sprinzl, M., Horn, C., Brown, M., Ioudovitch, A., and Steinberg, S. (1998). Compilation of tRNA sequences and sequences of tRNA genes. *Nucleic Acids Res* 26(1), 148-153.

S2. Mitchell, A., Chang, H.Y., Daugherty, L., Fraser, M., Hunter, S., Lopez, R., et al. (2015). The InterPro protein families database: the classification resource after 15 years. *Nucleic Acids Res* 43(Database issue), D213-221. doi: 10.1093/nar/gku1243.

S3. Lam, S.D., Dawson, N.L., Das, S., Sillitoe, I., Ashford, P., Lee, D., et al. (2016). Gene3D: expanding the utility of domain assignments. *Nucleic Acids Res* 44(D1), D404-409. doi: 10.1093/nar/gkv1231.

S4. Ashkenazy, H., Abadi, S., Martz, E., Chay, O., Mayrose, I., Pupko, T., et al. (2016). ConSurf 2016: an improved methodology to estimate and visualize evolutionary conservation in macromolecules. *Nucleic Acids Res* 44(W1), W344-350. doi: 10.1093/nar/gkw408.
